# AtPR5K2, a PR5-Like Receptor Kinase, Modulates Plant Responses to Drought Stress by Phosphorylating Protein Phosphatase 2Cs

**DOI:** 10.3389/fpls.2019.01146

**Published:** 2019-10-11

**Authors:** Dongwon Baek, Min Chul Kim, Dhinesh Kumar, Bokyung Park, Mi Sun Cheong, Wonkyun Choi, Hyeong Cheol Park, Hyun Jin Chun, Hee Jin Park, Sang Yeol Lee, Ray A. Bressan, Jae-Yean Kim, Dae-Jin Yun

**Affiliations:** ^1^Division of Applied Life Science (BK21plus program), Plant Molecular Biology and Biotechnology Research Center, Gyeongsang National University, Jinju, South Korea; ^2^Institute of Agriculture & Life Science, Gyeongsang National University, Jinju, South Korea; ^3^Donald Danforth Plant Science Center, St Louis, MO, United States; ^4^Gyeongsangnam-do Agricultural Research and Extension Services, Jinju, South Korea; ^5^Division of Ecological Conservation, Bureau of Ecological Research, National Institute of Ecology (NIE), Seocheon, South Korea; ^6^Department of Biomedical Science and Engineering, Konkuk University, Seoul, South Korea; ^7^Institute of Glocal Disease Control, Konkuk University, Seoul, South Korea; ^8^Department of Horticulture and Landscape Architecture, Purdue University, West Lafayette, IN, United States

**Keywords:** drought stress, abscisic acid, receptor-like kinase, ABI1, ABI2, SnRK2.6, phosphorylation, *Arabidopsis thaliana*

## Abstract

Cell surface receptors perceive signals from the environment and transfer them to the interior of the cell. The *Arabidopsis thaliana* PR5 receptor-like kinase (AtPR5K) subfamily consists of three members with extracellular domains that share sequence similarity with the PR5 proteins. In this study, we characterized the role of AtPR5K2 in plant drought-stress signaling. *AtPR5K2* is predominantly expressed in leaves and localized to the plasma membrane. The *atpr5k2-1* mutant showed tolerance to dehydration stress, while *AtPR5K2*-overexpressing plants was hypersensitive to drought. Bimolecular fluorescence complementation assays showed that AtPR5K2 physically interacted with the type 2C protein phosphatases ABA-insensitive 1 (ABI1) and ABI2 and the SNF1-related protein kinase 2 (SnRK2.6) proteins, all of which are involved in the initiation of abscisic acid (ABA) signaling; however, these interactions were inhibited by treatments of exogenous ABA. Moreover, AtPR5K2 was found to phosphorylate ABI1 and ABI2, but not SnRK2.6. Taken together, these results suggest that AtPR5K2 participates in ABA-dependent drought-stress signaling through the phosphorylation of ABI1 and ABI2.

## Introduction

Plant receptors perceive signals from external stimuli and transmit this information to the interior of the cell (McCarty and Chory, 2000). These receptors are typically composed of three major domains: an external ligand-binding domain for detecting the signal, a transmembrane domain for anchoring to the cell membrane, and an intracellular domain for transmitting the signal inside the cell to generate a signaling cascade (Hohmann et al., 2017). To sense and transmit the vast numbers of signals arising from environmental stimuli, plants have functionally evolved a large family of membrane receptor kinases and receptor-like kinases (RLKs) (Shiu and Bleecker, 2001; Hohmann et al., 2017). In the *Arabidopsis thaliana* genome, the RLKs are represented by 610 proteins divided into 44 subfamilies and at least 16 types, which have unique extracellular domain structures and functions (Shiu and Bleecker, 2001). Plant RLKs have different functions according to the types of motifs in their extracellular domains (Shiu and Bleecker, 2001); for example, the extracellular domains of leucine-rich repeat RLKs (LRR-RLKs) play important roles in the protein–protein interactions required for various signal transduction pathways in plant growth and development (Kobe and Deisenhofer, 1994; Torii et al., 1996; Clark et al., 1997; Yokoyama et al., 1998; Schoof et al., 2000; Mandel et al., 2016). The S-receptor kinases (SRKs) that possess a membrane-spanning serine/threonine kinase motif are involved in the determination of pollen-derived S-haplotype specificity for self-incompatibility (Hatakeyama et al., 2001; Takayama and Isogai, 2003). The lectin RLKs interact with extracellular carbohydrates such as glucose, mannose, fructose, chitobiose, and other sugars, and play roles in plant developmental processes and the signaling responses to plant hormones during various abiotic and biotic stresses (Loris et al., 2003; Vaid et al., 2012; Yang et al., 2016). The CRINKLY4 (CR4) family of RLKs contain a tumor necrosis factor receptor motif and are required for vegetative growth, floral organ development, aleurone formation in seeds, and sex determination (Becraft et al., 1996; Jin et al., 2000; Kang et al., 2002; Tian et al., 2007; Nikonorova et al., 2015), while the wall-associated kinase family of RLKs possess epidermal growth factor-like domains and are essential regulators of cell expansion, immunity resistance, and heavy metal tolerance in *Arabidopsis* (Verica and He, 2002; Verica et al., 2003; Hou et al., 2005; Wang et al., 2012). The pathogenesis-related 5 (PR5) RLKs (PR5Ks) are activated by several hormones and pathogenic infections (Thomma et al., 1998; Clarke et al., 2000; Durrant and Dong, 2004; van Loon et al., 2006).

Osmotin has been classified into the PR5 family with thaumatin-like domain (Abdin et al., 2011). Osmotin has been identified as the predominant protein (24 kDa protein) from osmotically adapted tobacco cells (Singh et al., 1985; Singh et al., 1987; Yun et al., 1997). In addition, osmotin is synthesized in root in response to exogenous abscisic acid (ABA) and accumulated in the presence of NaCl (Singh et al., 1987). The osmotin and osmotin-like proteins (OLPs), having antifungal activity, are basic isoform of the thaumatin-like proteins (TLPs) and share highly similar amino acid sequences (Yun et al., 1997; Shiu and Bleecker, 2001; Abdin et al., 2011; Misra et al., 2016). In addition to their common thaumatin-like domain, the OLPs and TLPs contain two additional domains to function, protein kinase-like domains and bifunctional inhibitor/lipid-transfer/seed storage 2S albumin domains (Liu J et al., 2010; Abdin et al., 2011). These proteins have been implicated in a wide range of cellular processes, including enzyme activation, the assembly of macromolecules, the cellular localization of proteins, and protein degradation (Liu J et al., 2010; Abdin et al., 2011). Moreover, the expression of OLP and TLP genes is induced by various environmental stresses, such as pathogens, salt, ABA, drought, cold, and wounding, suggesting that they may also function in stress signaling (Singh et al., 1989; Yun et al., 1998; van Loon et al., 2006; Misra et al., 2016). An *in silico* analysis of the structural features of these proteins suggested that they bind to specific receptors (Misra et al., 2016); however, the nature of their binding partners and the molecular and phenotypic consequences of such interactions remain unknown.

The phytohormone ABA is associated with diverse processes in plant growth and development, including seed maturation, seed dormancy, stomatal closure, and seedling growth (Finkelstein et al., 2002). ABA also plays major roles in plant-adaptive mechanisms to abiotic stresses such as cold, drought, and salinity, principally by regulating stomatal closure (Zhu, 2002; Raghavendra et al., 2010; Roychoudhury et al., 2013). The protein phosphatase type 2C (PP2C) proteins, including ABA-insensitive 1 (ABI1), ABI2, AtPP2CA/ABA hypersensitive germination 3 (AHG3), AHG1, hypersensitive to ABA 1 (HAB1), and HAB2, serve as negative regulators of ABA signaling (Kuhn et al., 2006; Saez et al., 2006; Yoshida et al., 2006b; Fujii et al., 2009a; Nishimura et al., 2009; Nishimura et al., 2010). In the absence of ABA, the activated PP2Cs bind to the SNF1-related protein kinase 2s (SnRK2s) and dephosphorylate their serine residues, preventing them from phosphorylating their targets in the ABA signaling pathway (Yoshida et al., 2006a; Fujii and Zhu, 2009b). ABA signals are detected by the pyrabactin resistance (PYR)/PYR1-like (PYL)/regulatory component of ABA receptor (RCAR) proteins, key ABA receptors that activate ABA signaling by binding both the ABA molecule and the PP2C proteins to inhibit the phosphatase activity of the PP2Cs (Fujii et al., 2009a; Park et al., 2009; Nishimura et al., 2010; Gonzalez-Guzman et al., 2012; Lee et al., 2013). This releases the SnRK2s from the PP2Cs–SnRK2s complex, which enables them to be activated by autophosphorylation and/or phosphorylation by other kinases (Melcher et al., 2009; Yin et al., 2009). The activated SnRK2s phosphorylate and activate several transcription factors involved in ABA signal transduction, such as the ABRE binding factor (ABF) transcription factors (Umezawa et al., 2009b; Xie et al., 2012). The core components, including the PYLs, PP2Cs, and SnRK2s, form a signaling complex known as the “ABA signalosome” or “ABA core signaling module” (Umezawa et al., 2009a and Umezawa et al., 2009b); however, the detailed regulation of the ABA signalosome in RLK-mediated signaling requires further studies.

In plant expressed sequence tag databases, 3 PR5Ks from among 27 proteins in the PR5 family included thaumatin domain in N-terminal region (Liu J et al., 2010). Here, we characterized the roles of the three *Arabidopsis* PR5-like receptor kinases (AtPR5Ks) in ABA-dependent drought-stress response. The *atpr5k2-1* mutant was found to be hypersensitive to drought stress but resistant to exogenous ABA stress. We revealed that AtPR5K2 acts as a negative regulator of ABA signaling during drought stress. AtPR5K2 phosphorylates ABI1 and ABI2 and functions most probably by modulating the phosphatase activity of PP2Cs of the ABA signalosome components.

## Materials and Methods

### *In silico* Analysis

The amino acid sequence analysis programs on TAIR (https://www.arabidopsis.org/) were used to search for homologous genes. Multiple sequence alignments of proteins were carried out using ClustalW (http://www.genome.jp/tools/clustalw) and the Plant Protein Phosphorylation DataBase (http://www.p3db.org). 

### Plant Materials and Growth Conditions

The *A. thaliana* wild type (WT) used in this study was Col-0 ecotype, and all mutant and gene-overexpressing transgenic plants had a Col-0 background. The *pr5k1-1* (Salk_142707), *pr5k2-1* (GK_321B01), and *pr5k3-1* (GABI_254G07) mutants were obtained from the *Arabidopsis* Biological Resource Center (*pr5k1-1*) and the GABI collection (*pr5k2-1* and *pr5k3-1*). Homozygous mutants were identified using a genomic PCR analysis with a T-DNA left border primer (LBb1.3) and two pairs of *PR5K*-specific primers. The sequences of experimental primers used for genotyping the various mutants are described in **Supplemental Table 1**.

*Arabidopsis* seeds were sterilized for 5 min with a 70% ethanol and 2% sodium hypochlorite solution (Yakuri pure chemicals, Kyoto, Japan) and then washed five times with sterilized water. After stratification for 3 days at 4°C in the dark, the seeds were plated on 1/2-strength Murashige and Skoog (MS) plates (pH 5.7) containing 0.6% agar and 1.5% sucrose, and grown in a growth chamber with 16-h light/8-h dark photoperiod at 23°C.

### Physiological Assays

To test drought sensitivity, 3-week-old plants grown in soil with sufficient water were not watered for 11 or 13 days. After rewatering, the recovery of the drought-treated plants was monitored. The drought experiments were repeated four times using at least 12 plants for each line in each experiment. To measure the transpirational water loss, leaves were detached from 4-week-old plants grown in soil and placed on Petri dishes. Their fresh weights were measured periodically at the indicated times and the percentage of water loss. The water loss assays were repeated three times using at least 15 plants for each line in each experiment.

To measure the percentage of cotyledon greening, the seeds of all experimental plants were harvested at the same time and grown on 1/2-strength MS plates (pH 5.7) containing 0.6% agar and 1.5% sucrose, without or with different concentrations of ABA (Sigma-Aldrich, St. Louis, MO, USA) and other abiotic stresses such as NaCl, mannitol, KCl, and LiCl. Cotyledon greening was determined after their expansion. The percentage of cotyledon greening was obtained from three biological replications using at least 48 seedlings for each line in each replication.

### Generation of *PR5K2*-Overexpressing Transgenic Plants

To generate the *PR5K2*-overexpressing transgenic plants, the full length of *PR5K2* complementary DNA (cDNA) was amplified from WT using PCR and cloned into *pMDC83* gateway vector (Thermo Fisher Scientific, MA, USA), which contained a hygromycin resistance gene and a *GFP* fusion sequence. *Arabidopsis* plants were transformed using *Agrobacterium tumefaciens*-mediated methods (Clough and Bent, 1998). The *PR5K2*-overexpressing transgenic plants were selected on 1/2 MS medium containing 30 μg/l of hygromycin (Merck, NJ, USA), and their *PR5K2* expression levels were analyzed using reverse transcription PCR (RT-PCR) using gene-specific primers listed in **Supplemental Table 1**.

### Quantitative RT-PCR Analysis

Total RNA was extracted and purified from different *Arabidopsis* tissues using the RNeasy Plant Mini Kit (Qiagen, Hilden, Germany), according to the manufacturer’s instructions, and treated with DNaseI (Sigma-Aldrich, St. Louis, MO, USA) to remove any genomic DNA contaminants. For the RT-PCR and quantitative RT-PCR (qRT-PCR) analyses, 2 µg total RNA was used for cDNA synthesis using SuperScript III (Thermo Fisher Scientific, MA, USA), in accordance with the manufacturer’s protocol. The qRT-PCR analysis was performed using a SYBR Green Supermix kit (Bio-Rad Laboratories, Hercules, CA, USA), and the relative gene expression levels were automatically calculated using the CFX384 real-time PCR detection system (Bio-Rad Laboratories, Hercules, CA, USA). The qRT-PCR was performed using the following conditions: 95°C for 10 min, followed by 50 cycles of 95°C for 10 s, 60°C for 30 s, and 72°C for 30 s. The expression of *TUBULIN2* was used as the endogenous control. The qRT-PCR experiments were performed in three independent replicates. The gene-specific primers used are listed in **Supplemental Table 1**.

### Transient Assays to Determine the Subcellular Localization of PR5K2

The full-length *PR5K2* cDNA was cloned into the *Xba*I and *Bam*HI sites of a superfolder green fluorescent protein (sGFP) vector plasmid containing the sGFP to create a chimeric GFP-fusion construct under the control of the CaMV *35S* promoter. To investigate the subcellular localization of PR5K2, *PR5K2-sGFP* was introduced into *Arabidopsis* protoplasts using a polyethylene glycol-mediated transformation (Baek et al., 2013). To confirm the localization of PR5K2 in the plasma membrane, the *pMDC83* vector containing *PR5K2-GFP* was transformed into *A. tumefaciens* (GV3101 strain). The transformed cells were infiltrated into the leaves of 3-week-old tobacco (*Nicotiana benthamiana*) plants. The *PR5K2-GFP*-infiltrated leaves were plasmolyzed by cutting them into small pieces and soaking them in an enzyme solution including osmoticum (mannitol and MgSO_4_) and a protectant (CaCl_2_) for 1 h. The fluorescent signals were detected using GFP filter (excitation, 488 nm; emission, 510 nm) and RFP filter (excitation, 543 nm; emission, 581 nm) on a confocal laser-scanning microscope (Olympus FV1000; Olympus, Tokyo, Japan). The wavelength range of bright field was in 30 nm for GFP and 100 nm for RFP. The confocal lasers were used argon for GFP and Green HeNe for RFP.

### Kinase Assays

The sequences of the PR5K2 kinase domain (wPR5K2KD) and the mutagenized PR5K2 kinase domain (mPR5K2KD) were amplified from full-length *PR5K2* using PCR and sequence-specific primers. The mPR5K2KD kinase domain was generated using site-directed mutagenesis methods that converted the lysine residue of the kinase domain to alanine. The *wPR5K2KD* and *mPR5K2KD* sequences were cloned into the *Eco*RI and *Sal*I sites of the *pGEX5X-1* vector, which provided the GST expression. The GST-fused recombinant proteins were expressed in *Escherichia coli* (BL21 strain) and purified using glutathione sepharose 4B (GE Healthcare, Chicago, IL, USA), according to the manufacturer’s instructions. GST proteins were used as a control. For the *in vitro* autophosphorylation assays, 2 μg of the recombinant protein was incubated at 30°C for 30 min in a kinase buffer containing 20 mM 4-(2-hydroxyethyl)-1-piperazineethanesulfonic acid (pH 7.5), 20 mM MgCl_2_, 2 mM MnCl_2_, and 1 μCi of [γ-32P] ATP (3,000 Ci/mmol). After separating the reaction products on a 10% polyacrylaminde gel using sodium dodecyl sulfate polyacrylamide gel electrophoresis (SDS-PAGE), the phosphorylated proteins were detected using autoradiography.

The in-gel kinase assays were performed using a 10% polyacrylaminde SDS-PAGE gel embedded with 0.1 mg/ml either ABI1 or ABI2 as a kinase substrate, as previously described (Liu X et al., 2010) and with minor modifications. The mutagenized ABI1 and ABI2 proteins were generated using site-directed mutagenesis methods that converted the serine residue of their kinase domains to alanine. After electrophoresis with GST, wPR5K2KD, or mPR5K2KD, the gels were washed three times with washing buffer containing 25 mM Tris–HCl (pH 7.5), 0.5 mM dithiothreitol (DTT), 0.1 mM Na_3_VO_4_, 5 mM NaF, 5% dried nonfat milk, and 0.1% Triton X-100. After removing the SDS, the gels were incubated at room temperature for 30 min in a reaction buffer containing 25 mM Tris–HCl (pH 7.5), 2 mM EGTA, 12 mM MgCl_2_, 1 mM DTT, and 0.1 mM Na_3_VO_4_. The reaction samples were combined with 250 nM ATP and 50 μCi of [γ-^32^P] ATP (3,000 Ci/mmol) in the same reaction buffer, then incubated at room temperature for 1.5 h. ABI1 and ABI2 phosphorylation were visualized using autoradiography.

### Bimolecular Fluorescence Complementation Assays in Tobacco Leaves

Bimolecular fluorescence complementation (BiFC) assays were performed using *Agrobacterium*-infiltrated methods (Tian et al., 2011). The full-length sequences of *PR5K2*, *ABI1*, *ABI2*, *SnRK2.6*, and *PYR1* were cloned into the binary gateway vectors *pDEST-* *^GW^* *VYNE* or *pDEST-* *^GW^* *VYCE* (Gehl et al., 2009). The N-terminal fragment (YFP^VN^; 1–173 a.a. of eYFP) of Venus eYFP was fused to PR5K2, while the C-terminal fragment (YFP^VC^; 156–239 a.a. of eYFP) was fused to the putative interaction partners used in the BiFC assay. The leaves of 4-week-old *N. benthamiana* plants were coinfiltrated with *A. tumefaciens* (OD_600_ = 0.5) carrying *pDEST-* *^GW^* *VYNE-PR5K2* (*PR5K2* *^VN^*) and either *pDEST-* *^GW^* *VYCE-ABI1* (*ABI1* *^VC^*), *pDEST-* *^GW^* *VYCE-ABI2* (*ABI2* *^VC^*), *pDEST-* *^GW^* *VYCE-SnRK2.6* (*SnRK2.6* *^VC^*), or *pDEST-* *^GW^* *VYCE-PYR1* (*PYR1* *^VC^*), together with the *p19* plasmid, in infiltration buffer (10 mM MES, 10 mM MgCl_2_, and 100 µM acetosyringone). After 2 days of incubation, the fluorescence signals were detected using a GFP filter (excitation, 488 nm; emission, 510 nm) on a confocal laser-scanning microscope (Olympus FV1000; Olympus, Tokyo, Japan).

### Co-Immunoprecipitation Assays

The full length of *ABI1* and *ABI2* cDNA was amplified from the WT using PCR and cloned into the *pEarleyGate 301* gateway vector (Thermo Fisher Scientific, Waltham, MA, USA), which contained a *Basta* resistance gene and a *HA* fusion sequence. The total proteins from equal amounts of *N. benthamiana* leaves expressing both *PR5K2-GFP* and *ABI1-HA* or *ABI2-HA* proteins were extracted in extraction buffer consisting of 100 mM Tris–HCl (pH 7.5), 150 mM NaCl, 1% NP-40, 1 mM ethylenediaminetetraacetic acid, 3 mM DTT, 2 mM Na_2_VO_3_, 2 mM NaF, 50 mM MG132, and protease inhibitor cocktail (Roche, Basel, Switzerland). α-GFP cross-linked to protein A agarose (Thermo Fisher Scientific, Waltham, MA, USA) was added to the total protein extract and incubated for 1 h at 4°C. After electrophoresis, immunoblotting was carried out using rat α-HA antibodies (Roche, Basel, Switzerland) and rabbit α-GFP antibodies (Abcam, Cambridge, UK). The antigen protein was detected using chemiluminescence with the enhanced-chemiluminescence-detecting reagent (GE Healthcare, Chicago, IL, USA) and ChemiDoc^™^ System (Bio-Rad Laboratories, Hercules, CA, USA).

## Results

### Identification of the AtPR5K2 Receptor Kinase Involved in Drought-Stress Signaling

**Figure 1:**
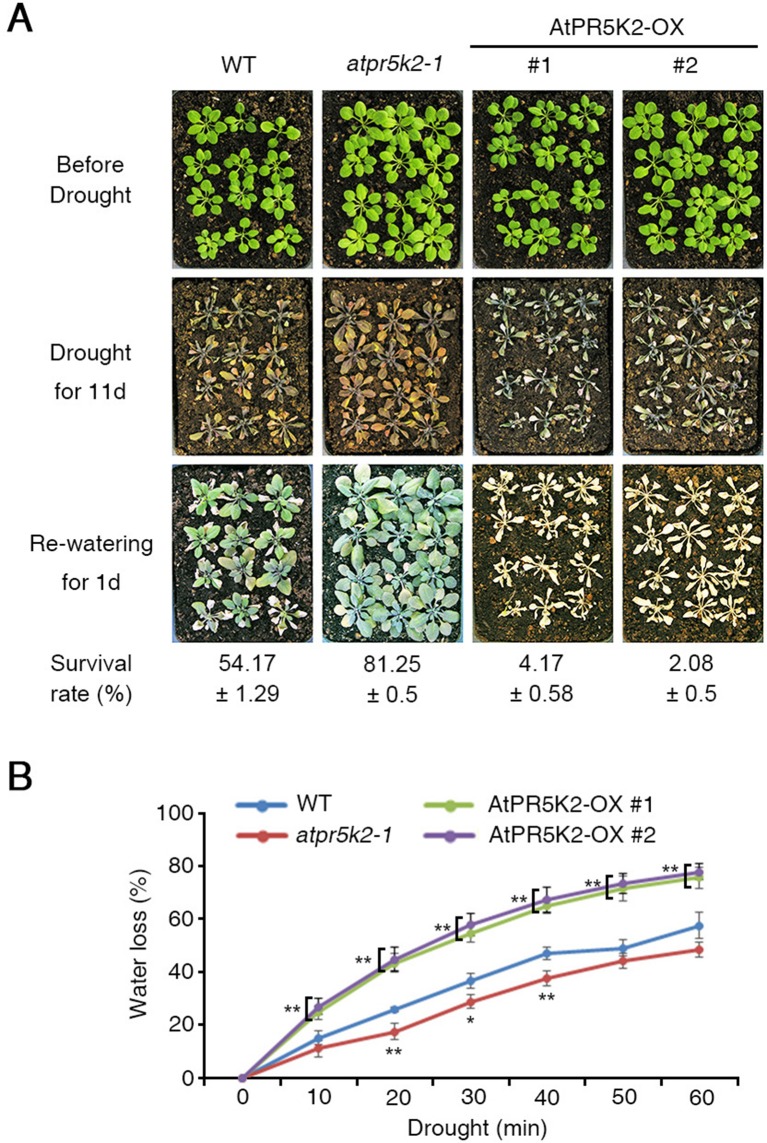
The phenotypes of the *atpr5k2-1* mutant and AtPR5K2-overexpressing (AtPR5K2-OX) plants in response to drought stress. **(A)** Wild type (WT), *atpr5k2-1*, and AtPR5K2-overexpressing plants (AtPR5K2-OX #1 and #2) were grown in soil with sufficient water for 3 weeks (upper row). Water was then withheld from the plants for 11 days (middle), after which the plants were rewatered for 1 day (bottom). The survival rates of the WT, *atpr5k2-1*, and AtPR5K2-OX #1 and #2 lines under drought conditions were assessed in four replicates (*n* = 48). **(B)** Transpirational water loss was measured in detached leaves of 4-week-old WT, *atpr5k2-1*, and AtPR5K2-OX #1 and #2 plants. The fresh weights were measured at the indicated time points, and water loss was normalized relative to a percentage of their initial fresh weight. Error bars indicate the standard deviation from three independent experiments. Asterisks represent significant differences from the WT (*0.01 < *p* ≤ 0.01, Student’s *t* test).

The PR5Ks, which exist in both monocots and dicots, are composed of a signal peptide, and a transmembrane domain, an extracellular thaumatin-like domain, and an intracellular Ser/Thr kinase domain (Shiu and Bleecker, 2001; Liu J et al., 2010; Abdin et al., 2011; **Supplementary Figure 1A**). There are three *Arabidopsis PR5K* genes: *AtPR5K1* (*At5g38280*), *AtPR5K2* (*At4g18250*), and *AtPR5K3* (*At1g70250*). The amino acid sequence of AtPR5K1 has a 55.0% similarity to AtPR5K2 and a 56.2% similarity to AtPR5K3, while the amino acid sequence of AtPR5K2 has a 50.6% similarity to AtPR5K3 (**Supplementary Figure 1B**). We analyzed expression of *PR5Ks* genes by *Arabidopsis* eFP Browser at BAR website (http://bar.utoronto.ca/efp/cgi-bin/efpWeb.cgi). Expression patterns of *PR5K1*, *PR5K2*, and *PR5K3* genes in developmental tissues were very different from each other (**Supplementary Figure 2**). To understand the biological function of the AtPR5Ks, we identified homozygous T-DNA insertion knockout mutants of the three *AtPR5K* genes, *atpr5k1-1*, *atpr5k2-1*, and *atpr5k3-1*, using genomic PCR and RT-PCR analyses (**Supplementary Figure 3**). These three *atpr5k* mutants did not show any significant phenotypic differences to the WT plants under normal growth conditions (**Figure 1A** and **Supplementary Figure 4A**).

We then tested the potential involvement of the *PR5K*s with the plant responses to various abiotic stresses, including drought stress. To test their drought tolerance, water was withheld from 3-week-old WT and *atpr5k* mutant plants for 11 days, after which they were rewatered. After 1 day of rewatering, a drought tolerance phenotype could be observed in the *atpr5k2-1* mutant plants (∼81.25% survival rate) in comparison with the WT plants (∼54.17% survival rate) (**Figure 1A**); however, the drought responses of the *atpr5k1-1* and *atpr5k3-1* mutants were similar to the WT plants. This suggests that *AtPR5K2* plays a role in drought-stress signaling (**Supplementary Figure 4**). In additions, the messenger RNA expression of *RD29B* in *atpr5k2-1* mutant was higher than that in WT plants under drought stress condition (**Supplementary Figure 5**). To further confirm the role of *AtPR5K2* in stress signaling, we generated AtPR5K2-overexpressing (AtPR5K2-OX) plants by introducing the full-length *AtPR5K2* cDNA into the WT plant. Two independent AtPR5K2-OX lines (#1 and #2) with different levels of *AtPR5K2* expression were selected (**Supplementary Figure 6**). When subjected to 11 days of drought stress and 1 day of rewatering, the AtPR5K2-OX plants exhibited a hypersensitivity to drought response (approximately 2.08–4.17% survival rate) than the WT plants (**Figure 1A**).

We further examined the transpirational water loss of the WT, *atpr5k2-1*, and AtPR5K2-OX plants by measuring the changes in the fresh weights of detached leaves from 4-week-old plants over time. Water was lost more slowly from the *atpr5k2-1* plants than from the WT, but more rapidly from the AtPR5K2-OX plants (**Figure 1B**). In addition, in the absence of ABA, stomatal opening in WT, *atpr5k2-1*, and AtPR5K2-OX plants were not different. However, in the presence of ABA, stomatal aperture in *atpr5k2-1* mutant was more significantly decreased than that in WT. The stomatal aperture of AtPR5K2-OX plants was comparable to that of WT (**Supplementary Figure 7**). We also tested the potential involvement of AtPR5K2 in the responses to other abiotic stresses; however, both the *atpr5k2-1* mutant and the AtPR5K2-OX plants did not show any obvious phenotypic differences to WT in response to the NaCl, KCl, LiCl, and mannitol stresses (**Supplementary Figure 8**). These results suggest that *AtPR5K2* plays a role in the regulation of plant responses to drought stress specifically.

### AtPR5K2 Plays a Negative Role in ABA Signaling

**Figure 2:**
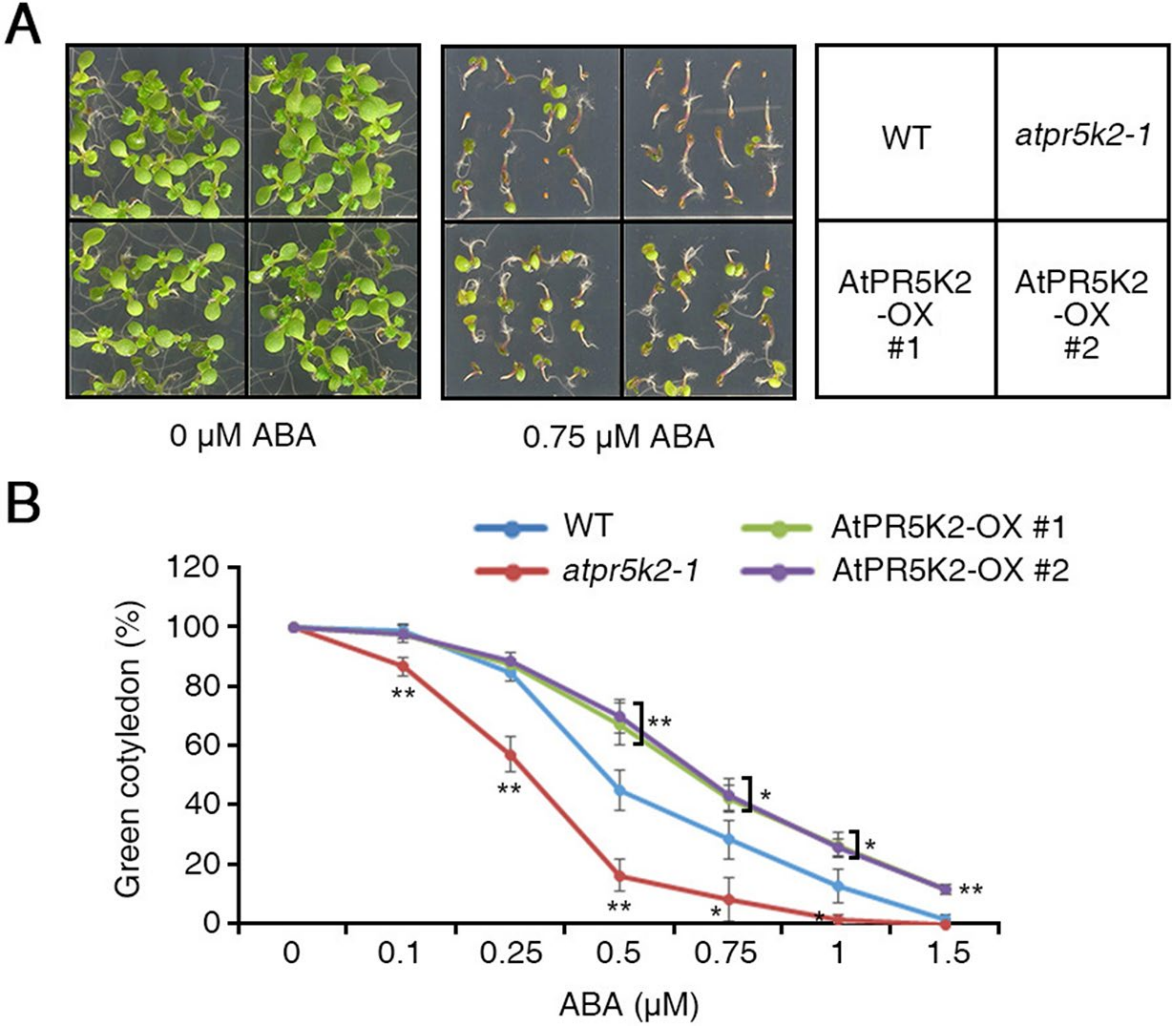
The phenotypes of the *atpr5k2-1* mutant and AtPR5K2-overexpressing (AtPR5K2-OX) plants in response to ABA stress. **(A)** WT, *atpr5k2-1*, and AtPR5K2-OX #1 and #2 plants were germinated on 1/2 Murashige and Skoog (MS) with and without 0.75 μM abscisic acid (ABA) for 5 days. The ABA sensitivity analysis was performed in triplicate by using at least 50 seeds from each line in each experiment. **(B)** The cotyledon greening of WT, *atpr5k2-1*, and AtPR5K2-OX #1 and #2 seedlings germinated on 1/2 MS containing different concentrations of ABA for 5 days. Cotyledon greening was determined as a percentage of the seeds plated (*n* = 50). Error bars indicate the standard deviation from three independent experiments. Asterisks represent significant differences from the WT (*0.01 < *p* ≤ 0.01, Student’s *t* test).

Water deficiency in plants has huge influences on plant growth and productivity (Bartels and Sunkar, 2005). Under water-deficient conditions, the initial plant response is to regulate the accumulation of ABA (Bartels and Sunkar, 2005). To determine whether AtPR5K2 is involved in ABA signaling, we examined the phenotypes of the *atpr5k2-1* mutant and AtPR5K2-OX plants when treated with exogenous ABA. When placed on a medium containing 0.75 µM ABA, the germination of the AtPR5K2-OX seeds was significantly enhanced compared with the WT, although the germination of the *atpr5k2-1* seeds was not affected (**Figure 2A**). After germination in the presence of 0.5 µM ABA, the *atpr5k2-1* mutants showed slower (∼2.7-fold) cotyledon greening than the WT; however, the cotyledon greening of the AtPR5K2-OX plants was ∼1.56-fold faster than that of the WT (**Figure 2B**). The results indicated that AtPR5K2 functions as a negative regulator of ABA signaling during seed germination.

### Functional Characterization of AtPR5K2

**Figure 3:**
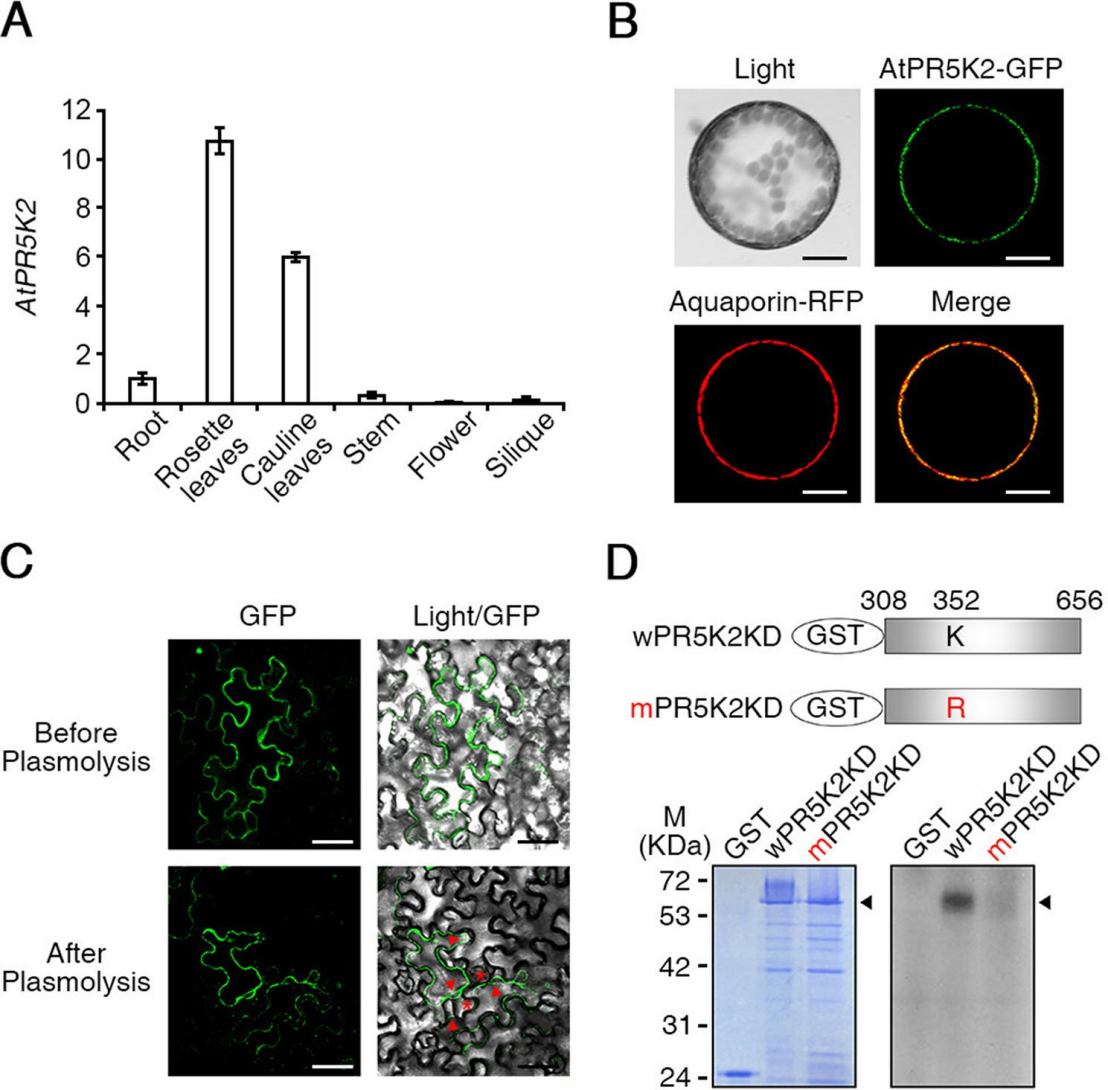
Expression patterns and subcellular localization of AtPR5K2. **(A)** The expression of *AtPR5K2* in various tissues of *Arabidopsis thaliana*. Total RNA was extracted from the roots, rosette leaves, cauline leaves, stems, flowers, and siliques of the wild-type plants. The transcript levels of *AtPR5K2* were measured using quantitative reverse transcription PCR (qRT-PCR) and calculated relative to the expression of the endogenous control gene, *TUBULIN2*. Error bars represent the ± SD from three independent experiments. **(B)** Subcellular localization of AtPR5K2 in *Arabidopsis* protoplasts. *35S:AtPR5K2-GFP* and *Aquaporin-RFP* were coexpressed in *Arabidopsis* protoplasts, which were analyzed using confocal fluorescence microscopy and photographed after 24 h of incubation at 22°C. Aquaporin-RFP is a plasma-membrane marker. Scale bars represent 10 μm. **(C)** Subcellular localization of AtPR5K2 in the epidermal cells of tobacco (*Nicotiana benthamiana*) leave expressing *35S:AtPR5K2-GFP* before and after plasmolysis. The epidermal cells were analyzed using confocal fluorescence microscopy and photographed after 48 h of incubation at 25°C. Scale bars represent 20 μm. Red asterisk indicates that AtPR5K2-GFP signal remains in the Hechtian strands. Red arrowheads point to the retracted plasma membrane. **(D)** *In vitro* kinase assays of AtPR5K2. The upper panel indicates the schematic structure of the GST-fused AtPR5K2 kinase domain (wPR5K2KD) and the GST-fused mutagenized AtPR5K2 kinase domain (mPR5K2KD). Each kinase domain was individually expressed in *Escherichia coli*, and 2 μg purified proteins was incubated in kinase assay buffer. Radioactive-labeled products were separated on sodium dodecyl sulfate polyacrylamide gel electrophoresis (SDS-PAGE) gels and detected using radioactivity (bottom right). After electrophoresis, the purified products were stained with Coomassie brilliant blue (bottom left).

To study the molecular functions of AtPR5K2, we used qRT-PCR to analyze the expression pattern of *AtPR5K2* in various *Arabidopsis* tissues, including the rosette leaves, roots, stems, cauline leaves, flowers, and siliques. We found that *AtPR5K2* is highly expressed in the rosette and cauline leaves (**Figure 3A**). Furthermore, the messenger RNA level of *AtPR5K2* was dramatically induced in response to drought stress (**Supplementary Figure 9**). To examine the subcellular localization of the AtPR5K2 protein, the full-length *AtPR5K2* cDNA was fused with GFP and expressed under the control of CaMV *35S* promoter. Aquaporin fused with red fluorescent protein was used as a plasma-membrane marker. *35S:AtPR5K2-GFP* and *35S:Aquaporin-RFP* were transiently coexpressed in *Arabidopsis* protoplasts, and the GFP signal was observed to overlap with the plasma-membrane signal, suggesting that the protein is localized to the plasma membrane (**Figure 3B**). To confirm its plasma membrane localization, we induced plasmolysis in tobacco epidermal cells expressing *AtPR5K2-GFP*. The GFP fluorescence was still observed at clear separation of the plant protoplast from the cell wall organizing Hechtian strands, which was interrelated cell wall and plasma membrane after plasmolysis (**Figure 3C**). These data indicated that AtPR5K2 was localized to the plasma membrane.

One of the AtPR5Ks, AtPR5K1, was previously been shown to have autocatalytic kinase activity *in vitro* (Wang et al., 1996). To determine whether AtPR5K2 has autophosphorylation activity, we fused the protein kinase domain-encoding sequence of *AtPR5K2* (wPR5K2KD) with *GST* and expressed it in *E. coli*. The purified recombinant wPR5K2KD proteins were reacted with radioactive [γ-^32^P] ATP and separated using SDS-PAGE. A phosphorylated band was detected on the resulting gel at the position approximating a molecular weight of 65 kDa, which corresponded to the molecular weight of the recombinant wPR5K2KD protein (**Figure 3D**). To impede the kinase activity of AtPR5K2, we substituted the lysine residue (amino acid position 352) in the kinase domain with arginine. This lysine residue is known to be important for ATP binding in many kinases (Carrera et al., 1993). The kinase activity of the mPR5K2KD protein was significantly reduced compared with that of wPR5K2KD (**Figure 3D**), indicating that AtPR5K2 has a functional kinase activity.

### AtPR5K2 Interacts With Protein Phosphatase 2C and SnRK2.6 in the ABA Signaling Pathway

**Figure 4:**
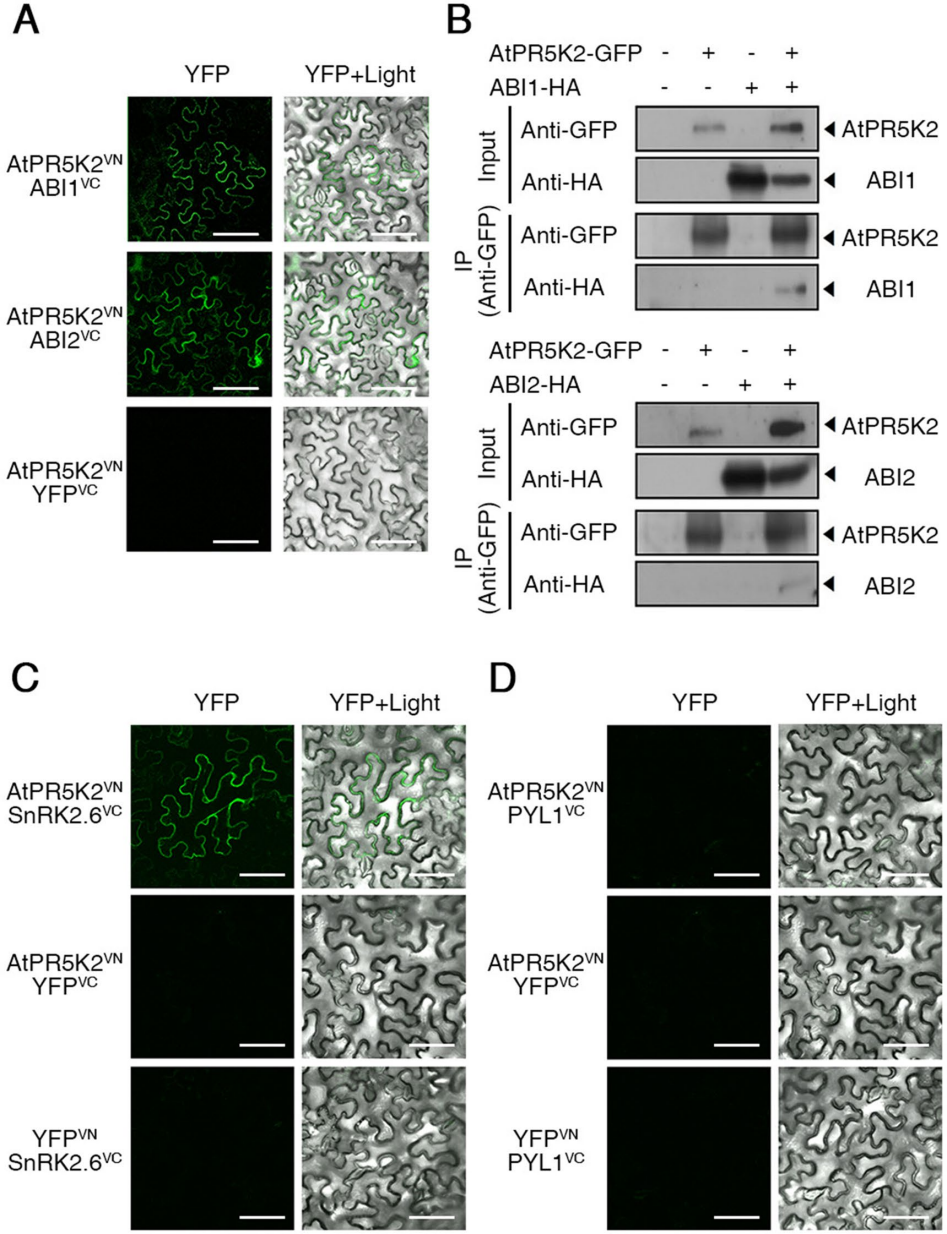
AtPR5K2 interaction with ABI1, ABI2, and SnRK2.6 *in vivo* and *in vitro*. **(A)** Bimolecular fluorescence complementation (BiFC) analysis of AtPR5K2 and PP2Cs coexpressed in tobacco (*Nicotiana benthamiana*) leaves. VN and VC indicate the N- and C-terminal regions of Venus (eYFP), respectively. The epidermal cells were analyzed using confocal fluorescence microscopy and photographed after 48 h of incubation at 25°C. Scale bars represent 100 μm. **(B)** AtPR5K2 forms a complex with the PP2Cs. Each combination of *35S:AtPR5K2-GFP*, *35S:ABI1-HA*, and *35S:ABI2*-HA were transiently expressed in tobacco plants. The proteins were immunoprecipitated with an alpha-green fluorescent protein (α-GFP) antibody and resolved with SDS-PAGE. The immunoblots were probed with an α-GFP antibody to detect AtPR5K2 or an α-HA antibody to detect ABI1 and ABI2. The minus (−) indicated empty vectors (*35S:GFP* or *35S:HA*, respectively) as negative controls. (C and D) BiFC analysis of AtPR5K2 and SnRK2.6 **(C)** or PYL1 **(D)** coexpressed in tobacco leaves. The epidermal cells were analyzed using confocal fluorescence microscopy and photographed after 48 h of incubation at 25°C. Scale bars represent 100 μm.

The core components of the ABA signalosome, including the PYR/PYL/RCAR ABA receptors, PP2Cs, and SnRK2s, play a major role in the ABA signal transduction and plant adaptive responses to environmental stresses (Ma et al., 2009; Melcher et al., 2009). The PP2Cs, ABI1 and ABI2, act as negative regulators of ABA signaling (Kuhn et al., 2006; Saez et al., 2006), while SnRK2.6/OST1 acts as a positive regulator of ABA-dependent stomatal closure (Yoshida et al., 2006a; Kulik et al., 2011). To test whether AtPR5K2 is involved in ABA signalosome-mediated signaling, we tested the *in vivo* interaction between AtPR5K2 and the PP2Cs using BiFC assays. The full-length *AtPR5K2* cDNA sequence was fused to the N-terminal fragment of the Venus protein (AtPR5K2^VN^), while ABI1 or ABI2 was fused to the C-terminal fragment of Venus (ABI1^VC^ or ABI2^VC^, respectively). Fluorescence signals were detected in the plasma membrane of the tobacco epidermal cells when AtPR5K2^VN^ was coexpressed with ABI1^VC^ or ABI2^VC^ (**Figure 4A**), while no fluorescence signals were detected when these constructs were coexpressed with the empty YFP^VC^ or YFP^VN^ vectors (**Supplementary Figure 10**). To further confirm the interaction of AtPR5K2 with ABI1 or ABI2, we conducted *in vivo* coimmunoprecipitation assays in tobacco leaves. The full-length AtPR5K2 sequence was fused to GFP (AtPR5K2-GFP), while ABI1 and ABI2 were individually fused to the HA tag protein (ABI1-HA or ABI2-HA). The total proteins of tobacco leave coinfiltrated AtPR5K2-GFP and ABI1-HA or ABI2-HA were immunoprecipitated with an α-GFP antibody, and the eluted precipitates were detected using an α-HA antibody, revealing that AtPR5K2 interacts with ABI1 and ABI2 *in vivo* (**Figure 4B**).

The physical interaction of the PP2Cs with the PYLs or SnRK2s was previously identified during ABA signaling (Yoshida et al., 2006a; Park et al., 2009; Nishimura et al., 2010). To test interaction between AtPR5K2 and SnRK2.6 or PYL1, we performed BiFC assays using SnRK2.6 or PYL1 fused to the C-terminal fragment of Venus (SnRK2.6^VC^ and PYL1^VC^, respectively). Fluorescence signals were detected in the plasma membrane of the epidermal cells coexpressing *AtPR5K2* *^VN^* and *SnRK2.6* *^VC^* (**Figure 4C**); however, no interaction was detected between AtPR5K2^VN^ and PYL1^VC^ (**Figure 4D**). Taken together, these results suggest that AtPR5K2 plays an important role in the regulation of ABA core signaling by interacting with the PP2Cs and SnRK2.6.

### ABA Affects the Interactions of AtPR5K2 With the PP2Cs and SnRK2.6

**Figure 5:**
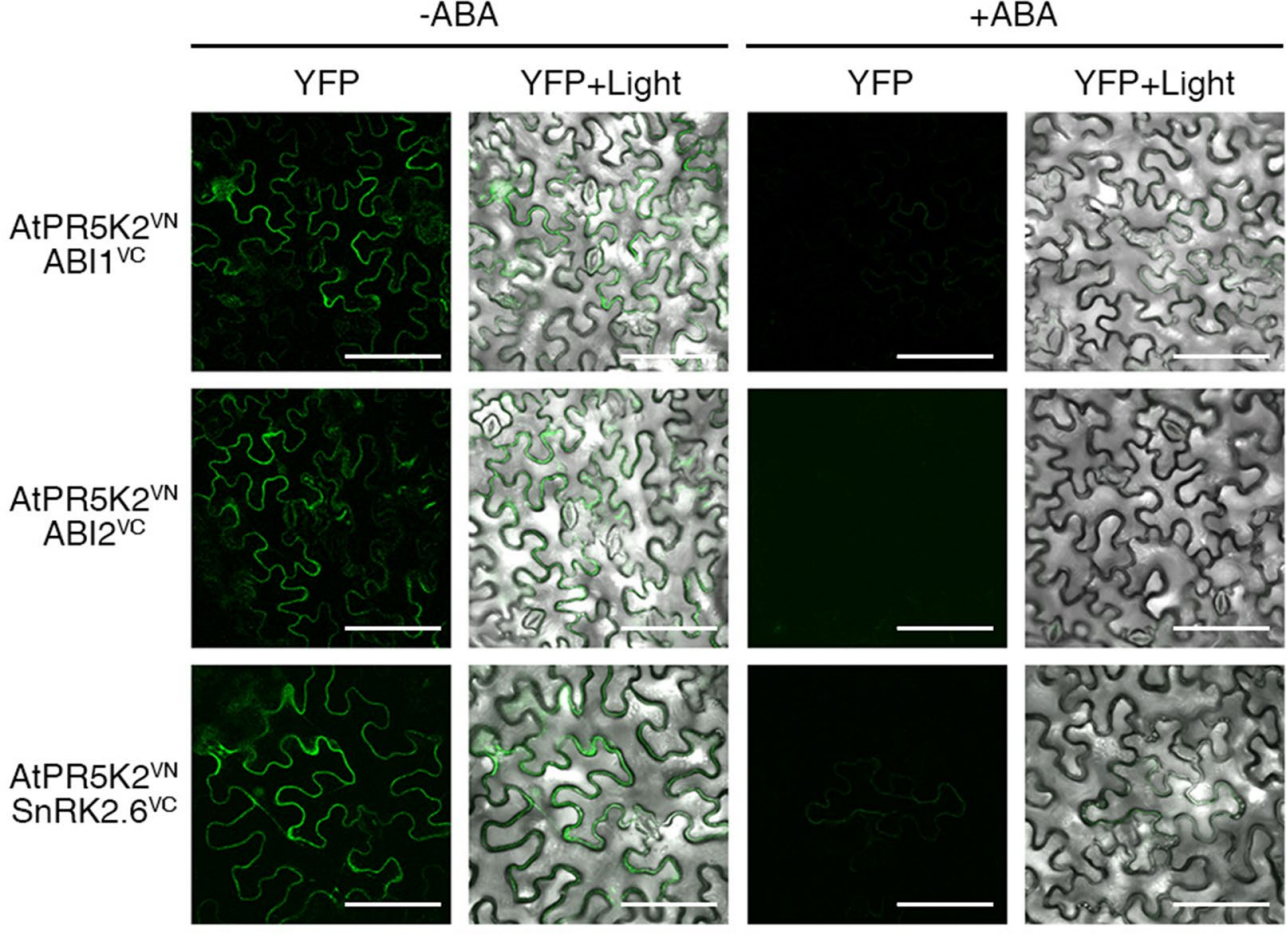
Influence of the AtPR5K2 complex on ABA stress signaling. BiFC analysis of AtPR5K2 and ABI1, ABI2, or SnRK2.6 transiently coexpressed in tobacco (*Nicotiana benthamiana*) leaves in the presence of absence of 10 µM exogenous ABA. VN and VC indicate the N- and C-terminal regions of Venus (eYFP), respectively. The epidermal cells were analyzed using confocal fluorescence microscopy and photographed after 48 h of incubation at 25°C in the presence or absence of ABA. Scale bars represent 100 μm

In the absence of ABA, the PP2Cs inhibit the autophosphorylation and activation of SnRK2.6 in ABA core signaling, while intracellular ABA enhances the *in vivo* interaction between the PP2Cs and PYL receptors (Nishimura et al., 2010). Thus, in the presence of ABA, the inhibitory effect of the PP2Cs on SnRK2.6 is removed, activating SnRK2.6 to phosphorylate ion channels or AREB/ABF transcription factors to stimulate the ABA response (Yoshida et al., 2006a; Yoshida et al., 2010). To investigate whether elevated cellular ABA levels affect the interaction of AtPR5K2 with the PP2Cs or SnRK2.6, we performed the BiFC assays in the presence and absence of exogenous ABA, by coexpressing AtPR5K2^VN^ with ABI1^VC^, ABI2^VC^, or SnRK2.6^VC^ in tobacco cells. The fluorescence signals in the tobacco leaves coexpressing AtPR5K2^VN^/ABI1^VC^, AtPR5K2^VN^/ABI2^VC^, and AtPR5K2^VN^/SnRK2.6^VC^ were weaker in the presence of 10 μM exogenous ABA conditions than in the absence of ABA (**Figure 5**), suggesting that elevated ABA levels disrupt the interaction of AtPR5K2 with ABI1, ABI2, and SnRK2.6.

### AtPR5K2 Phosphorylates the PP2Cs ABI1 and ABI2

**Figure 6:**
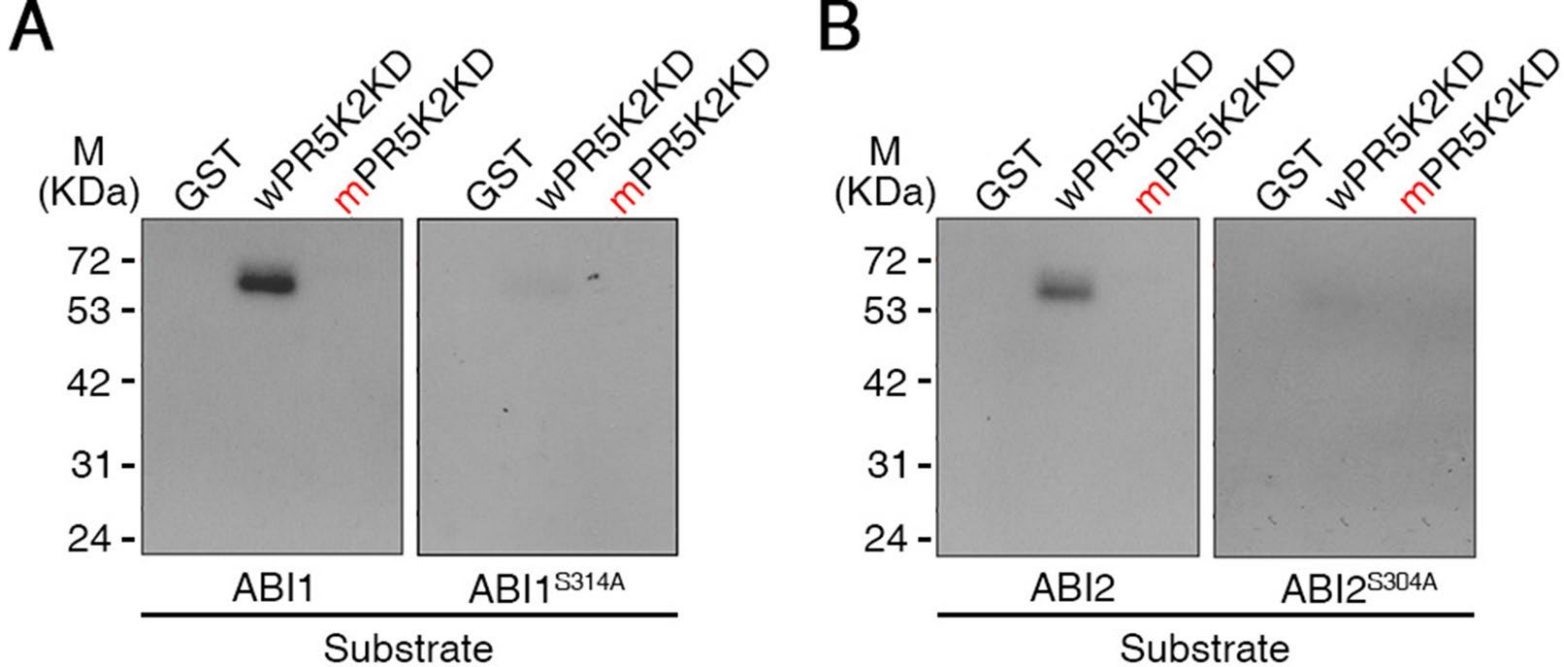
In-gel kinase assay of AtPR5K2. The recombinant fusion protein of the AtPR5K2 kinase domain (wPR5K2KD) or the mutagenized AtPR5K2 kinase domain (mPR5K2KD) was denatured and separated on an SDS gel. An in-gel kinase assay was performed using recombinant ABI1 (**A**; left), ABI1^S314A^ (**A**; right), ABI2 (**B**; left), or ABI2^S304A^ (**B**; right) as an embedded substrate. The GST tag served as a negative control.

To investigate whether AtPR5K2 could specifically phosphorylate ABI1, ABI2, or SnRK2.6, we performed in-gel kinase assays to test the phosphorylation of ABI1-GST, ABI2-GST, and SnRK2.6-GST by AtPR5K2. The kinase domain of PR5K2 (wPR5K2KD) phosphorylated ABI1 and ABI2 (**Figure 6**); however, no phosphorylation was detected when SnRK2.6 or GST were used as substrates (**Figure 6** and **Supplementary Figure 11**). In addition, the phosphorylation of ABI1 and ABI2 was strongly attenuated by the mutation of the PR5K kinase domain (mPR5K2KD; **Figure 6**).

To further confirm that ABI1 and ABI2 function as substrates for AtPR5K2, we investigated the putative phosphorylation site of ABI1 and ABI2 by analyzing the amino acid sequences the of PP2Cs using the Plant Protein Phosphorylation DataBase (http://www.p3db.org). The putative phosphorylation sites in ABI1 (Ser-314) and ABI2 (Ser-304) were substituted with alanine (ABI1^S314A^ and ABI2^S304A^, respectively; Han et al., 2018). We performed in-gel kinase assays using ABI1^S314A^ and ABI2^S304A^ as substrates for the AtPR5K2 kinase domain (**Figure 6**), revealing that their phosphorylation by wPR5K2KD was greatly attenuated in comparison with the WT ABI1 and ABI2 proteins (**Figure 6**). These results suggested that AtPR5K2 phosphorylates the ABI1 and ABI2 phosphatases to modulate their activities during ABA core signaling.

## Discussion

### AtPR5K2 Negatively Regulates ABA Signaling

Several RLKs are known to function in abiotic stress signaling (Osakabe et al., 2005; Bai et al., 2009; Tanaka et al., 2012; Kumar et al., 2017). Some RLKs are involved in ABA signaling; however, the mechanisms by which they interact with the ABA core signaling pathway were not previously understood. *AtRPK1* expression is rapidly induced by various abiotic stresses, such as ABA, drought, salinity, and cold, indicating that RPK1 mediates abiotic stress responses (Hong et al., 1997), while proline-rich extensin-like receptor kinase 4, an ABA- and Ca^2+^-activated protein kinase, was functionally characterized in the initial stages of ABA signaling (Bai et al., 2009). A cysteine-rich repeat RLK36negatively regulates ABA and osmotic stress signaling by interacting with receptor-like cytosolic kinase 1 (Tanaka et al., 2012). BRI1-associated receptor kinase 1 interacts with SnRK2.6 to induce stomatal closure in response to ABA, in contrast with the function of ABI1 during the ABA responses (Shang et al., 2016). Receptor dead kinase 1 is a positive regulator of ABA-dependent abiotic-stress signaling (Kumar et al., 2017). In the present study, we characterized the role of AtPR5K2 in ABA-dependent drought-stress signaling. It was found to interact with ABI1 and ABI2 on the plasma membrane (**Figure 4**) and phosphorylate them *in vitro* (**Figures 3** and **6**). ABI1 and ABI2 are typical PP2C proteins and key negative regulators of the ABA core signaling pathway (Cutler et al., 2010; Raghavendra et al., 2010). In addition, the AtPR5K2-OX plants underwent significantly higher levels of cotyledon greening than the WT when treated with ABA (**Figure 2**). These findings suggested that AtPR5K2 could act as a negative regulator of ABA signaling that modulates the functions of the PP2Cs.

Age-dependent leaf senescence is associated with various environmental stresses and changes of various plant hormones, such as ABA (Lim et al., 2007). It is recently reported that drought tolerance is enhanced when drought-induced leaf senescence is delayed in plants (Rivero et al., 2007). *Arabidopsis* RPK1, receptor protein kinase 1, mediates ABA-induced and age-dependent leaf senescence (Lee et al., 2011). The ABA receptor PYL9 improved ability of drought resistance as well as ABA-induced leaf senescence (Zhao et al., 2016). These results suggest the crosstalk between ABA-dependent drought stress signaling and leaf senescence. Based on these observations, it would be possible that AtPR5K2 may play a role in cross-talk between ABA-dependent drought stress signaling and leaf senescence. We intend to test the role of AtPR5K2 in drought- and ABA-mediated leaf senescence in the future study.

### AtPR5K2 Directly Phosphorylates the PP2Cs But Not SnRK2.6 in the ABA Signalosome

Although AtPR5K1 was previously reported to have kinase activity (Wang et al., 1996), the function of the closely related AtPR5K2 protein was not previously known. AtPR5K2 comprises a thaumatin-like domain, a transmembrane domain, and a Ser/Thr kinase domain, the latter of which showed high similarity with that of AtPR5K1 (**Supplementary Figure 1**). Like AtPR5K1, AtPR5K2 has an efficient kinase activity which requires the lysine residue in its kinase domain (**Figure 3D**).

We investigated several major components of the ABA core signaling pathway as potential target substrates for AtPR5K2. ABI1, ABI2, and SnRK2.6 were previously known to be activated by phosphorylation during the ABA response (Kulik et al., 2011; Ma et al., 2009; Umezawa et al., 2009b; Ng et al., 2011); however, the mechanisms of this phosphorylation remained unclear. We showed that AtPR5K2 phosphorylated ABI1 and ABI2 *in vitro* (**Figure 6**), but did not use SnRK2.6 as a substrate (**Supplementary Figure 11**). In addition, we identified the putative phosphorylation sites of ABI1 (Ser-314) and ABI2 (Ser-304) used by AtPR5K2 (**Figure 6**). To our knowledge, AtPR5K2 is the first plant novel receptor kinase known to phosphorylate ABI1 and ABI2.

### Proposed Working Model of AtPR5K2 in ABA Core Signaling

**Figure 7:**
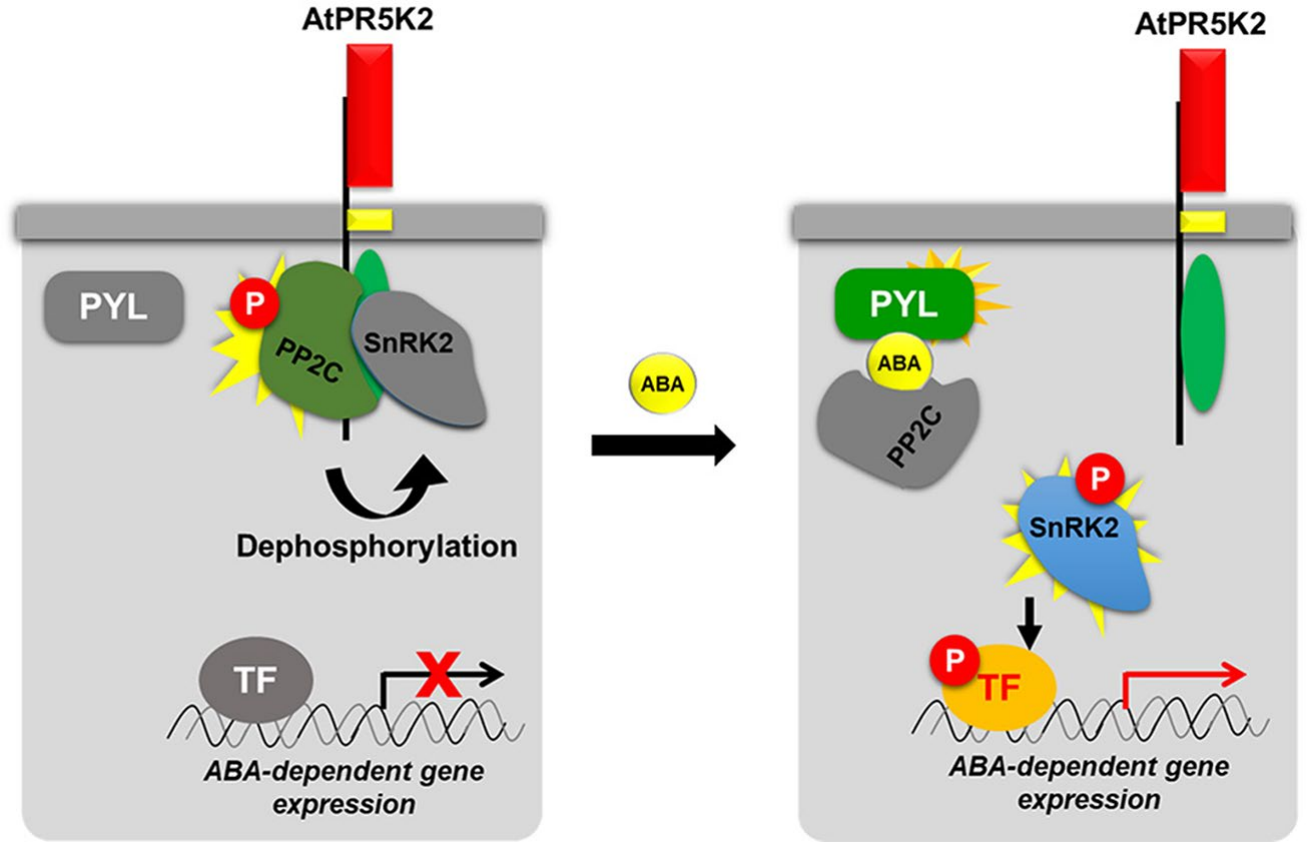
Proposed working model of AtPR5K2 in ABA core signaling. AtPR5K2 is single transmembrane protein with an N-terminal thaumatin-like domain (red box), a single transmembrane domain (yellow box), and a C-terminal Ser/Thr kinase domain (green oval). In the absence of ABA, the phosphatase activity of the PP2Cs is enhanced by AtPR5K2-dependent phosphorylation. The activated PP2Cs dephosphorylate the SnRK2s, decreasing their activity. Thus, the ABA-dependent gene expression regulated by SnRK2 is attenuated. In the presence of ABA, the protein–protein interaction affinities among AtPR5K2, PP2Cs, and SnRK2s are weakened. This means that the PP2Cs and SnRK2 are released from AtPR5K2, freeing SnRK2.6 to autophosphorylate itself and phosphorylate the transcription factors regulating ABA-dependent gene expression. In the case of the PP2Cs and SnRK2.6, a vivid coloration indicates an active status, while the gray color indicates an inactive status. The red circle containing the letter “P” indicates phosphorylation.

The ABA-mediated signal transduction pathway has been extensively studied, and many of its regulators have been elucidated (Finkelstein et al., 2002; Cutler et al., 2010; Raghavendra et al., 2010). Here, we added a new key component, AtPR5K2, to the ABA core signaling pathway (**Figure 7**). In the absence of ABA, AtPR5K2 interacts with the PP2Cs and SnRK2.6, but not with PYL1 ABA receptor. Active AtPR5K2 phosphorylates PP2C phosphatases, such as ABI1 and ABI2, which inhibit the activity of SnRK2.6, a positive regulator of ABA signaling, *via* dephosphorylation. This means that, in the absence of ABA, AtPR5K interrupts the ABA signal transduction and the expression of the ABA-dependent genes. Under stress conditions, such as drought, increased intracellular ABA dissociates the PP2Cs and SnRK2.6 from AtPR5K2 (**Figure 5** and **Figure 7**). The released PP2Cs directly bind to both ABA and the PYL receptors and become inactivated. Subsequently, the SnRK2.6 kinase is activated by autophosphorylation and phosphorylates the ABRE/ABF transcription factors to induce the expression of ABA-dependent genes, which enhances plant tolerance to various abiotic stresses (**Figure 7**).

## Conclusion

We identified and characterized the role of AtPR5K2, a PR5-like receptor kinase, in ABA core signaling during plant responses to drought stress. The AtPR5K2-OX plants were hypersensitive to drought stress and tolerant of exogenous ABA, suggesting that AtPR5K2 mediates ABA-dependent drought-stress signaling. Our molecular and biochemical results suggest that, under normal growth conditions, AtPR5K2 probably deactivates ABA core signaling and ABA-dependent gene expression by modulating the phosphorylation status of the PP2Cs and SnRK2.6, a major factor in stomatal closure. In response to abiotic stresses, however, AtPR5K2 turns on the ABA core signaling cascade and ABA-dependent gene expression by releasing the PP2Cs and freeing SnRK2.6 to promote ABA signaling. Our results demonstrate that AtPR5K2 plays an important role as a key negative regulator of ABA core signaling by phosphorylating the PP2C phosphatases, such as ABI1 and ABI2.

## Data Availability Statement

All datasets for this study are included in the manuscript and the Supplementary Files.

## Author Contributions

DB, MK, J-YK, and D-JY designed the experiments. DB and DK performed most of the experiments, and MK, J-YK, and D-JY wrote the manuscript. HC, SL, and RB discussed and commented on the results and manuscripts. BP, MC, WC, HCP, and HJP performed some of the experiments. D-JY, J-YK, MK, and DB provided funding for research work.

## Funding

This work was supported by the Next Generation BioGreen21 Program [SSAC, grant number PJ01318201 (to D-JY) and PJ01318202 (to MCK)], the Rural Development Administration Republic of Korea, and the Basic Science Research Program through the National Research Foundation of Korea (NRF) funded by the Ministry of Education [2015R1A6A1A03031413 (to MCK), NRF-2017R1A4A1015515 (to J-YK), 2016R1D1A1B01011803 (to DB), and Global Resea rch Laboratory 2017K1A1A2013146 (to D-JY)].

## Conflict of Interest

The authors declare that the research was conducted in the absence of any commercial or financial relationships that could be construed as a potential conflict of interest.
