# Supplementary figures and images for: AtPR5K2, a PR5-Like Receptor Kinase, Modulates Plant Responses to Drought Stress by Phosphorylating Protein Phosphatase 2Cs
Source: Front Plant Sci. 2019 Oct 11;10:1146. doi: 10.3389/fpls.2019.01146 (PMC6822995; doi:10.3389/fpls.2019.01146)

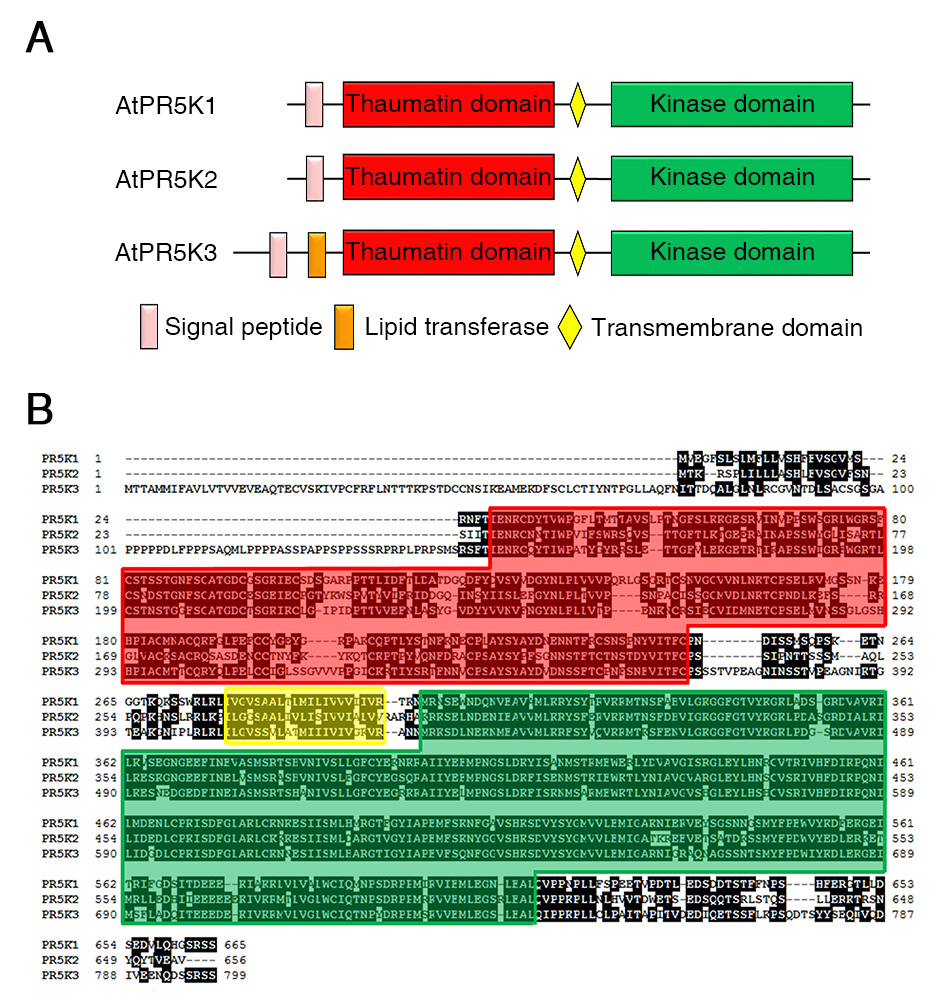

Supplement: Supplementary file 1 [file Image_1.jpeg]

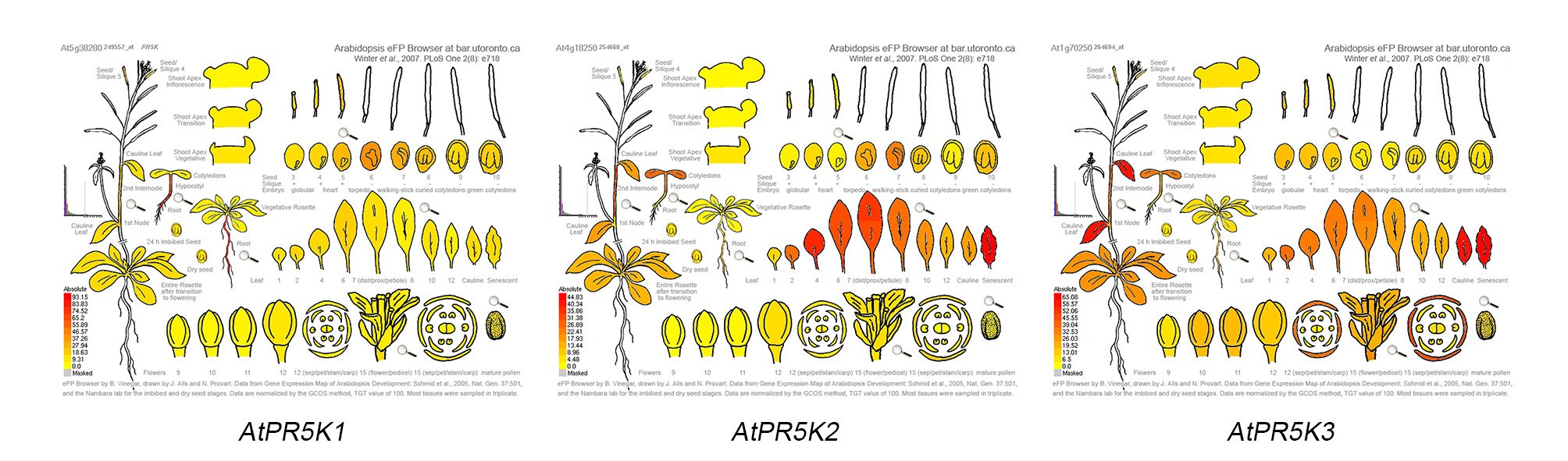

Supplement: Supplementary file 2 [file Image_2.jpeg]

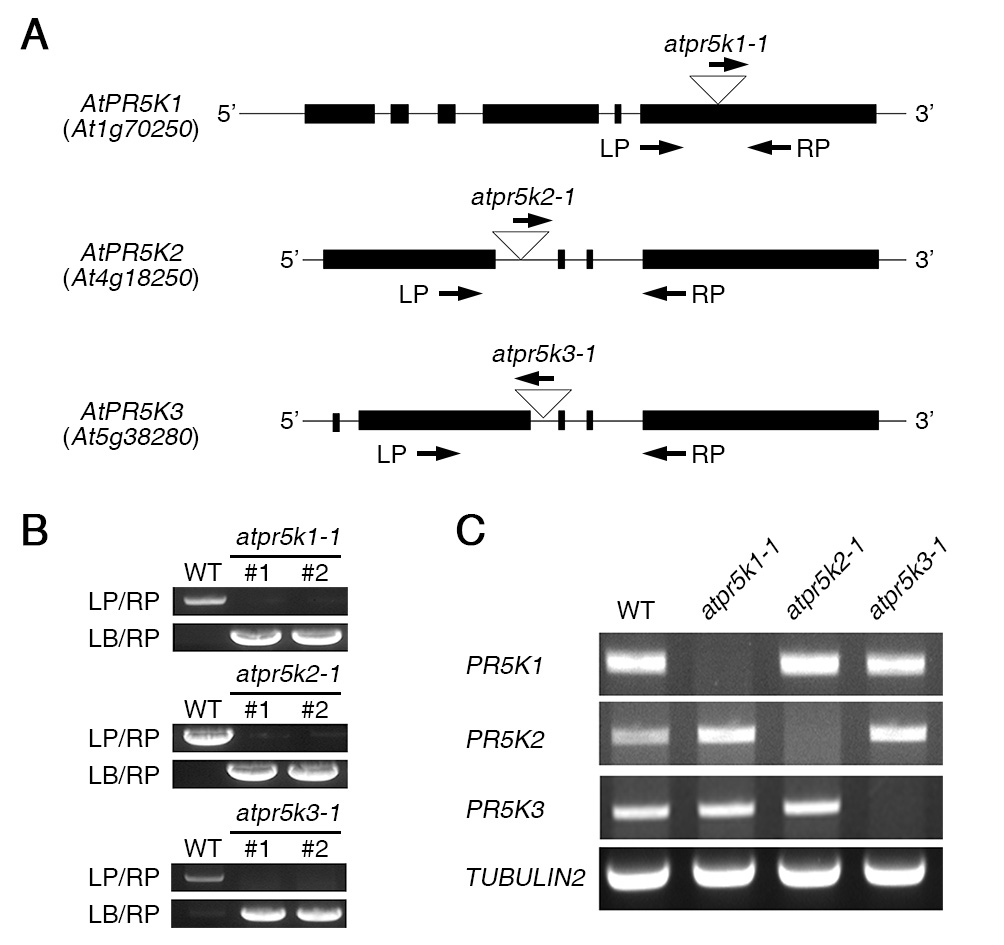

Supplement: Supplementary file 3 [file Image_3.jpeg]

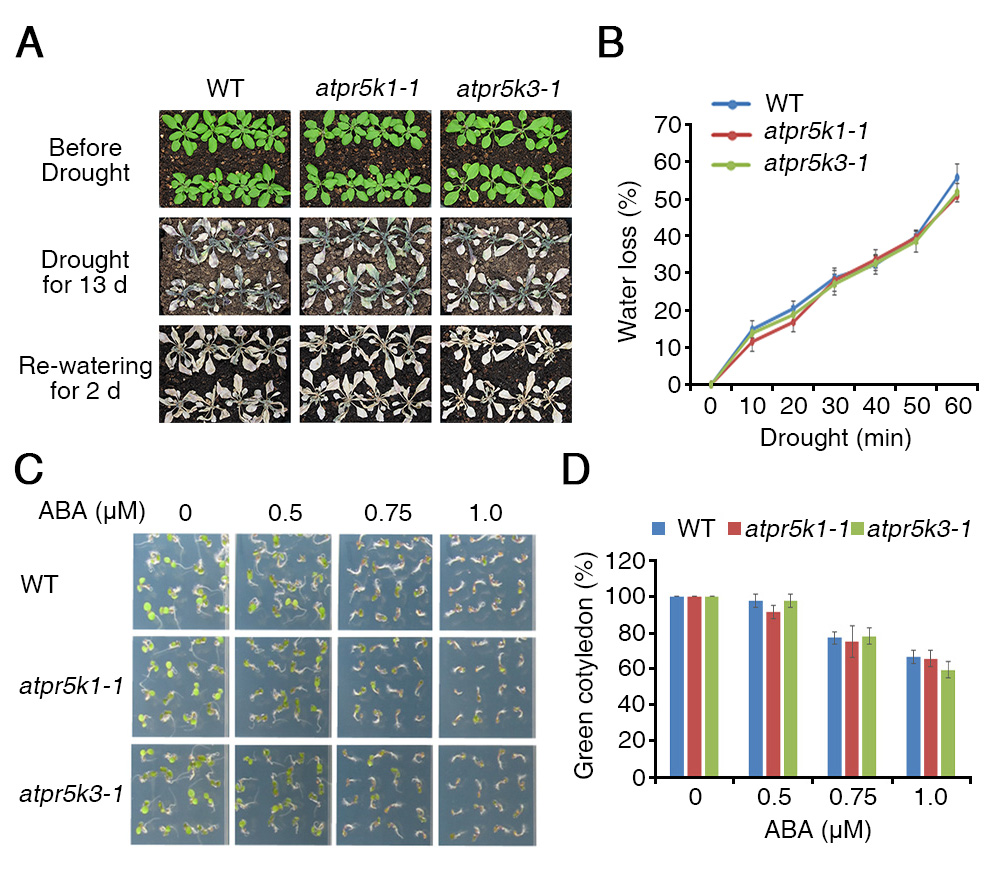

Supplement: Supplementary file 4 [file Image_4.jpeg]

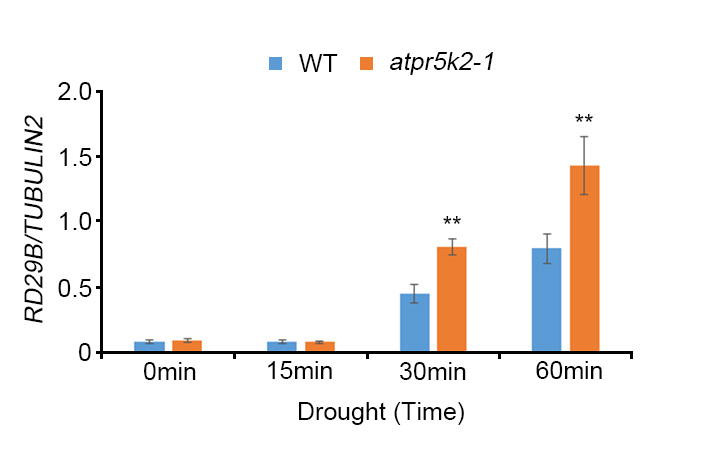

Supplement: Supplementary file 5 [file Image_5.jpeg]

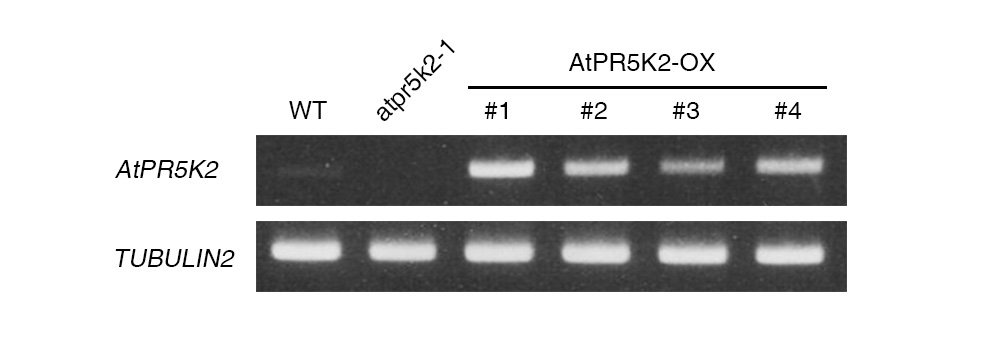

Supplement: Supplementary file 6 [file Image_6.jpeg]

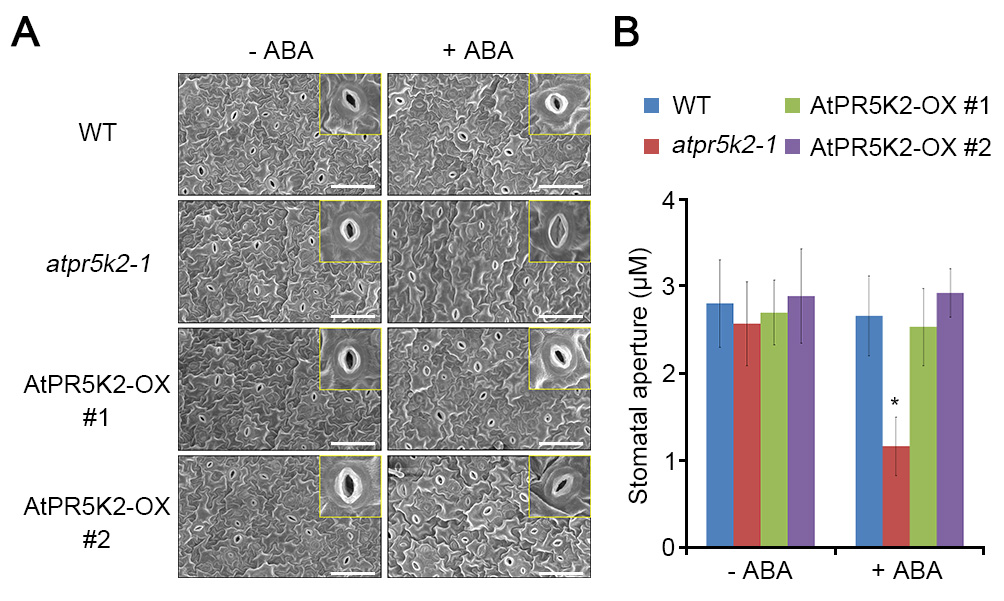

Supplement: Supplementary file 7 [file Image_7.jpeg]

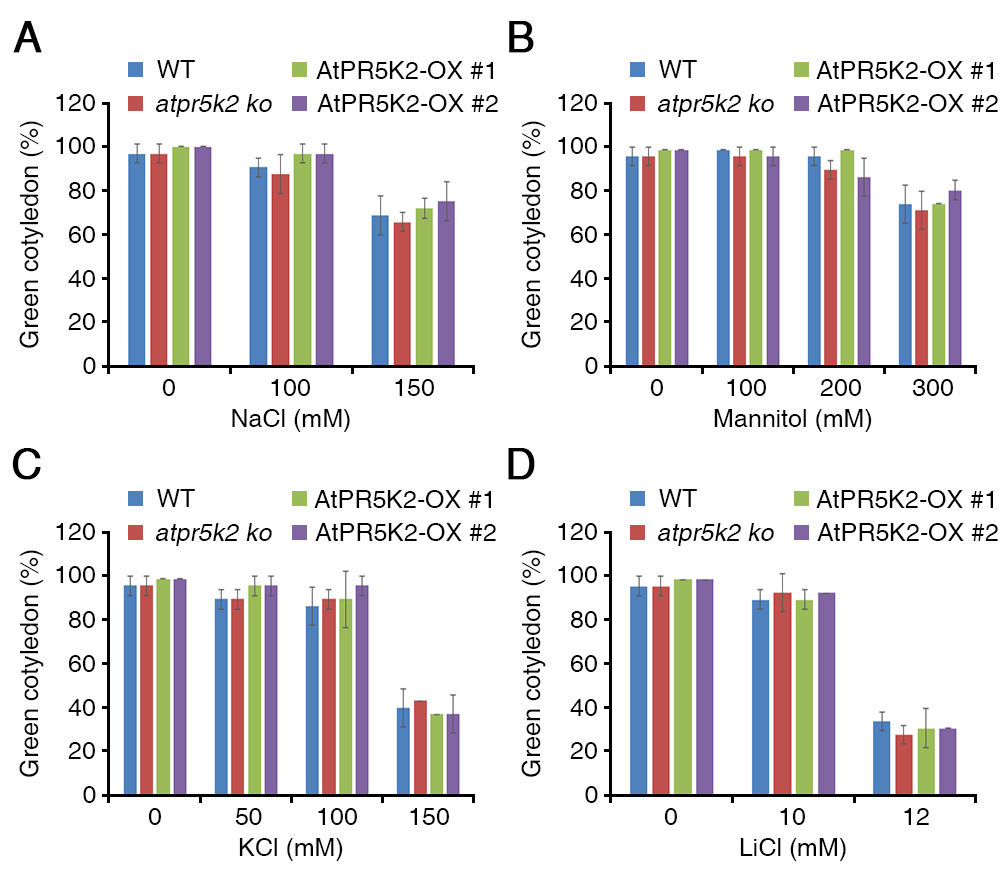

Supplement: Supplementary file 8 [file Image_8.jpeg]

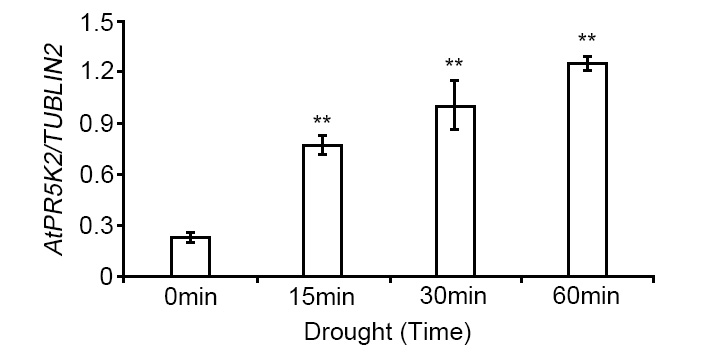

Supplement: Supplementary file 9 [file Image_9.jpeg]

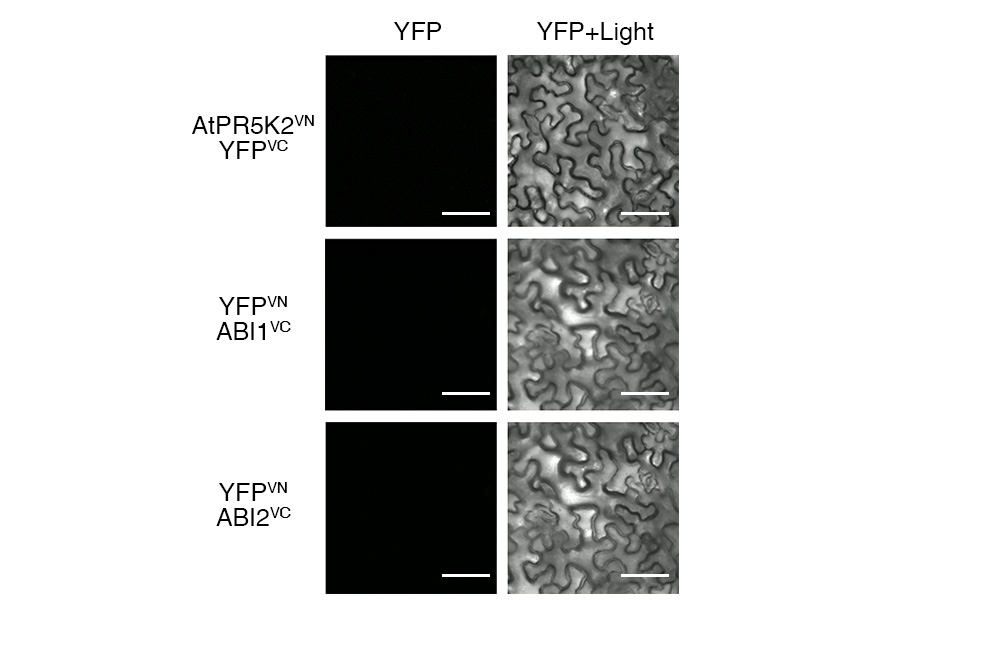

Supplement: Supplementary file 10 [file Image_10.jpeg]

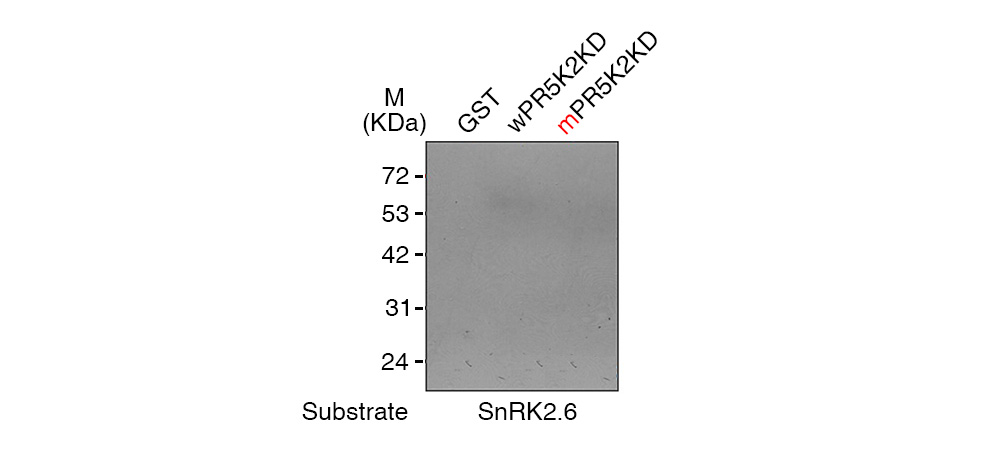

Supplement: Supplementary file 11 [file Image_11.jpeg]
