# Supplementary material for: AtPR5K2, a PR5-Like Receptor Kinase, Modulates Plant Responses to Drought Stress by Phosphorylating Protein Phosphatase 2Cs
Source: Front Plant Sci. 2019 Oct 11;10:1146. doi: 10.3389/fpls.2019.01146 (PMC6822995; doi:10.3389/fpls.2019.01146)
Supplement: Supplementary file 12 [file Table_1.docx]

***Supplementary Material***

**Title: AtPR5K2, a PR5-Like Receptor Kinase, Modulates Plant Responses to Drought Stress by Phosphorylating Protein Phosphatase 2Cs**

Dongwon Baek^a,^ †, Min Chul Kim^a, b,^ †, Dhinesh Kumar^a, c,^ †, Bokyung Park^d^, Mi Sun Cheong^e^, Wonkyun Choi^f^, Hyeong Cheol Park^f^, Hyun Jin Chun^b^, Sang Yeol Lee^a^, Ray A. Bressan^g^, Jae-Yean Kim^a,^ *, Dae-Jin Yun^d,^ *

* Corresponding author:

Department of Biomedical Science and Engineering, Konkuk University, Seoul 05029, Korea Phone: +82 2 450 0583

E-Mail: Jae-Yean Kim (kimjy@gnu.ac.kr), Dae-Jin Yun ([djyun@konkuk.ac.kr](mailto:djyun@konkuk.ac.kr))

† These authors contributed equally to this work.

**1. Supplementary Figures and Tables**

**1. 1. Supplementary Figures**


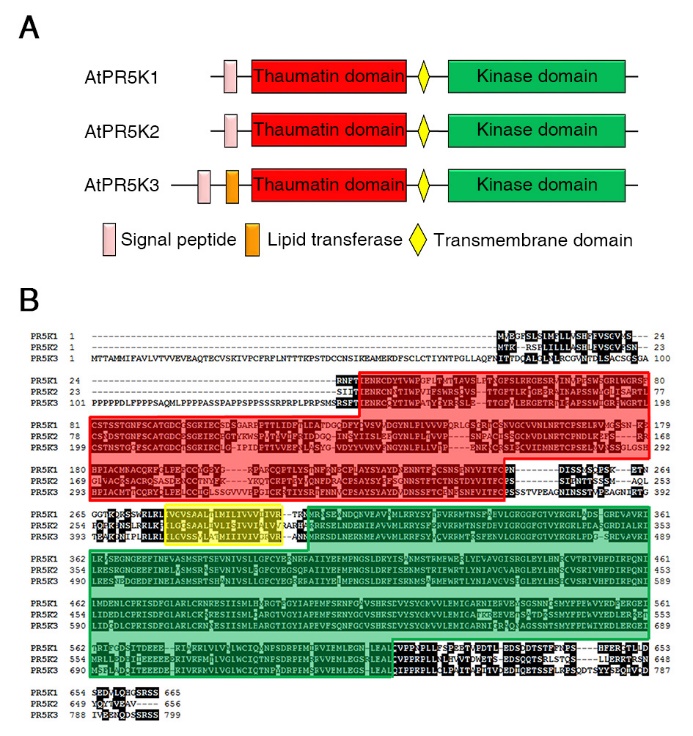


**Supplementary Figure 1.** Structural organization of the AtPR5K proteins.

(A) Conserved domains of the AtPR5Ks. (B) Alignment of the amino acid sequences of the AtPR5Ks. The gene locus numbers of the individual sequences are as follows: AtPR5K1, *At5g38280*; AtPR5K2, *At4g18250*; AtPR5K3, *At1g70250*, respectively.


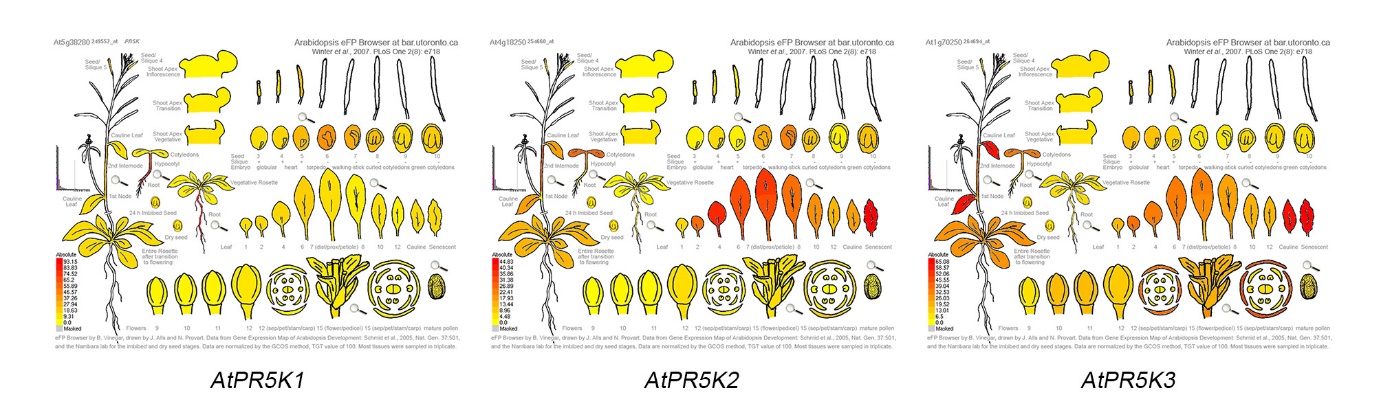


**Supplementary Figure 2.** Comparison of expression patterns of *AtPR5K1*, *AtPR5K2*, and *AtPR5K3*.

Expression patterns of *AtPR5K1*, *AtPR5K2*, and *AtPR5K3* in developmental tissues using *Arabidopsis* eFP Browser at BAR website (<http://bar.utoronto.ca/efp/cgi-bin/efpWeb.cgi>).


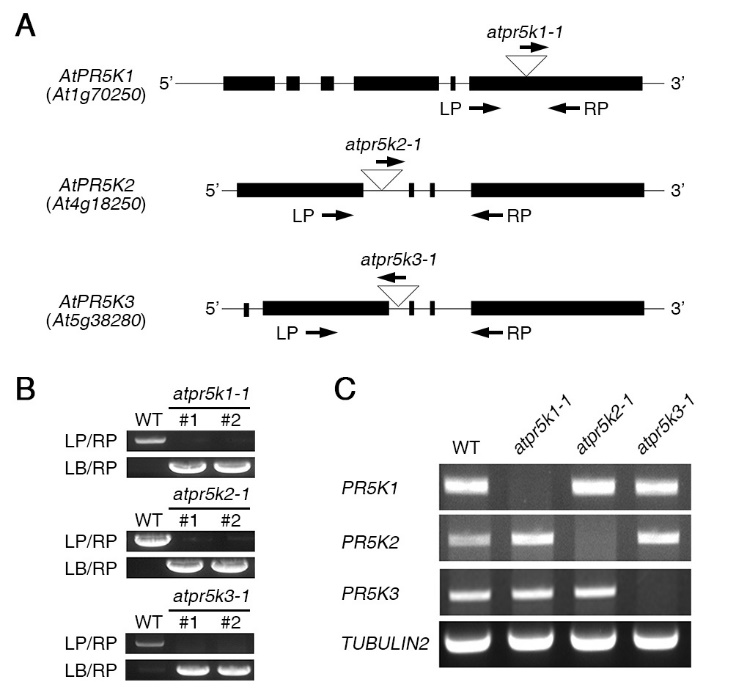


**Supplementary Figure 3.** Identification of *atpr5k1-1*, *atpr5k2-1*, and *atpr5k3-1* mutants.

(A) Scheme of the *AtPR5K1*, *AtPR5K2*, and *AtPR5K3* genes and the T-DNA insertions in the respective mutant lines. Boxes indicate exons, while lines indicate introns. LP and RP are gene-specific primers described in Supplementary Table 1, which were used for the diagnostic PCR analysis. (B) Genotyping of the *atpr5k1-1*, *atpr5k2-1*, and *atpr5k3-1* mutants using a diagnostic PCR analysis. LB indicates left border primer specific to the T-DNA. WT, wild type. (C) RT-PCR analysis of *AtPR5K1*, *AtPR5K2*, and *AtPR5K3* expression in each mutant. PCR reactions were performed using the gene-specific primers described in Supplementary Table 1. *TUBULIN2* was used as a loading control.


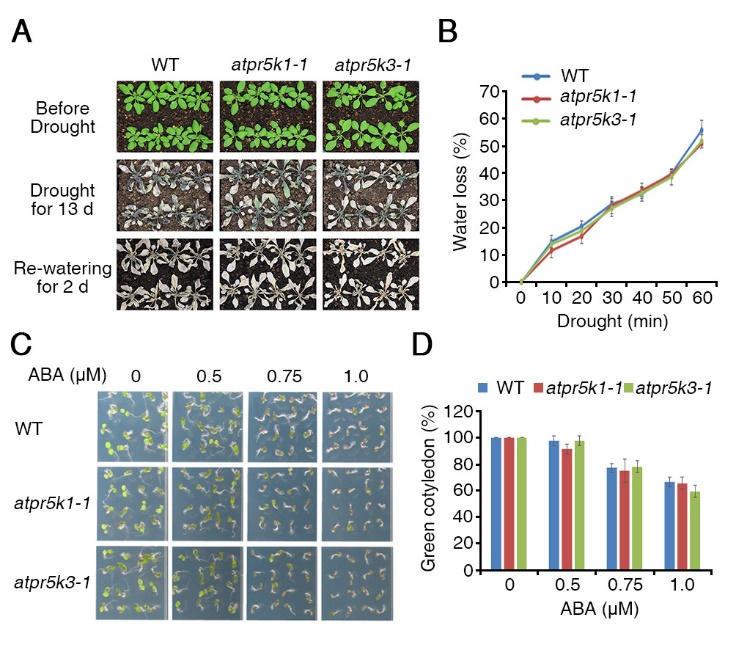


**Supplementary Figure 4.** The phenotypes of the *atpr5k1-1* and *atpr5k3-1* mutants in response to drought and ABA stresses.

(A) Wild-type (WT), *atpr5k2-1*, and *atpr5k3-1* plants were grown in soil with sufficient water for three weeks (upper panel). Water was then withheld from the plants for 13 days (middle), after which the plants were re-watered for one day (bottom). The survival rates of the WT, *atpr5k2-1*, and *atpr5k3-1* plants under drought conditions were assessed from four replicates (n = 48). (B) Transpirational water loss was measured in four-week-old WT, *atpr5k2-1*, and *atpr5k3-1* plants. The fresh weights were measured at the indicated time points, and water loss was normalized relative to a percentage of their initial fresh weight. Error bars indicate the standard deviation from three independent experiments. (C) WT, *atpr5k2-1*, and *atpr5k3-1* plants were germinated on 1/2 MS containing different concentrations of ABA for five days, after which their phenotypes were photographed. The ABA sensitivity analysis were performed in triplicate by using at least 50 seeds from each line in each experiment. (D) The cotyledon greening of the WT, *atpr5k2-1*, and *atpr5k3-1* seedlings depicted in (C) was assessed. Cotyledon greening was determined as a percentage of the seeds plated (n = 50). Error bars indicate the standard deviation from three independent experiments.


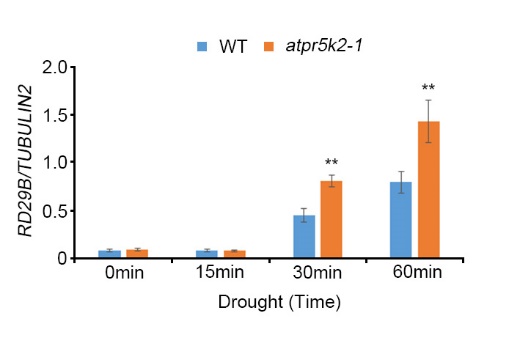


**Supplemental Figure 5.** Expression of *RD29B* in WT and *atpr5k2-1* plants under drought stress condition.

The ten-day-old seedlings of WT and *atpr5k2-1* mutant grown on 1/2 MS medium under long-day condition (Light 16 h/Dark 8 h). Total RNA extracted from WT and *atpr5k2-1* seedlings over one-hour dehydration. The expression of *TUBULIN2* was used as an internal control for normalization. Error bars represent the ±SD from three independent experiments. Asterisks represent significant differences from the value of 0min time (**, p-value ≤ 0.01, Student’s *t*-test).


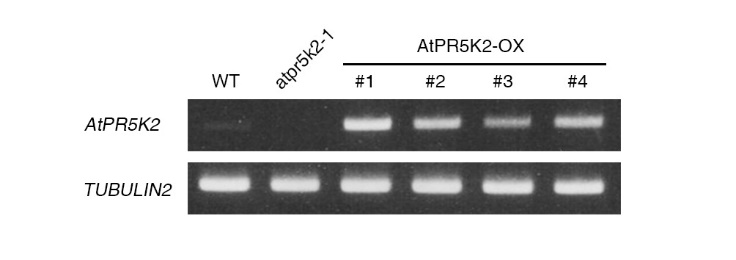


**Supplementary Figure 6.** Expression of *AtPR5K2* in AtPR5K2-OX plants.

RT-PCR analysis of WT, *atpr5k2-1*, and *AtPR5K2* overexpressing transgenic plants (AtPR5K2-OX). PCR reactions were performed using the gene-specific primers described in Supplementary Table 1. *TUBULIN2* was used as a loading control.


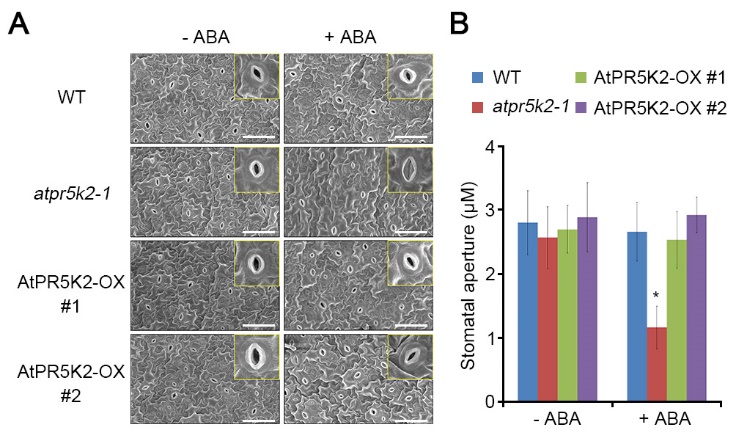


**Supplementary Figure 7.** AtPR5K2 mediated stomatal closure in response to exogenous ABA.

(A) Stomatal closure before and after exogenous ABA treatment was assessed in the leaf epidermis of WT, *atpr5k2-1* and AtPR5K2-OX plants. Leaves of ten-day-old WT, *atpr5k2-1*, and AtPR5K2-OX plants were incubated in stomatal opening solution for 2 h and treated to 1/2 MS liquid medium without (-ABA; 0 µM ABA) or with (+ABA; 5 µM ABA) ABA for 2 h. Stomatal closure in the leaf epidermis were observed using scanning microscopy. Scale bar indicates 50 µm. (B) Measurement of stomatal aperture (width/length) in WT, *atpr5k2-1* and AtPR5K2-OX plants before and after ABA treatment. At least 50 guard cells from each sample were measured. Error bars represent the SD from three independent experiments. Asterisks represent significant differences from the WT (*, 0.01 <p-value ≤ 0.01, Student’s t-test).


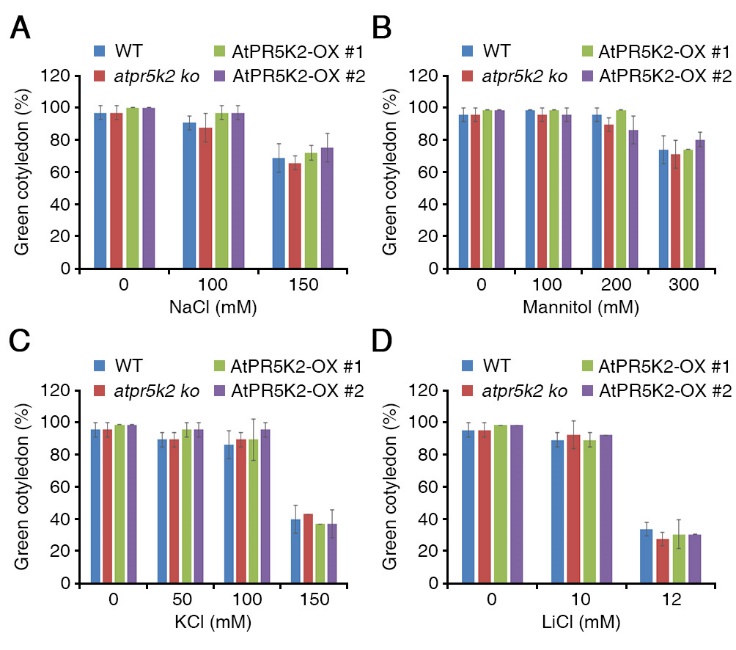


**Supplementary Figure 8.** The phenotypes of *atpr5k2-1* mutant and AtPR5K2-OX plants in response to various abiotic stresses.

Wild-type (WT), *pr5k2-1*, and AtPR5K2-OX (#1 and #2) plants were germinated on 1/2 MS containing different concentrations of NaCl (A), mannitol (B), KCl (C), and LiCl (D) for five days. Cotyledon greening was determined as a percentage of the seeds plated (n = 100). Error bars indicate the standard deviation from three independent experiments.


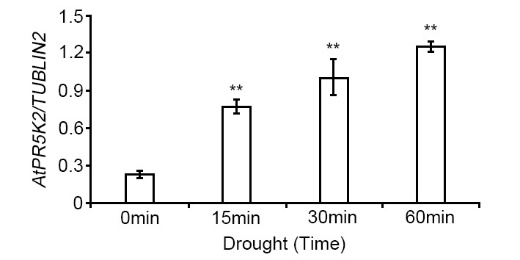


**Supplemental Figure 9.** Expression of PR5K2 in response to drought stress.

The ten-day-old WT seedling grown on 1/2 MS medium under long-day condition (Light 16 h/Dark 8 h). Total RNA extracted from WT plants over one-hour dehydration. The expression of *TUBULIN2* was used as an internal control for normalization. Error bars represent the ±SD from three independent experiments. Asterisks represent significant differences from the value of 0min time (**, p-value ≤ 0.01, Student’s *t*-test).


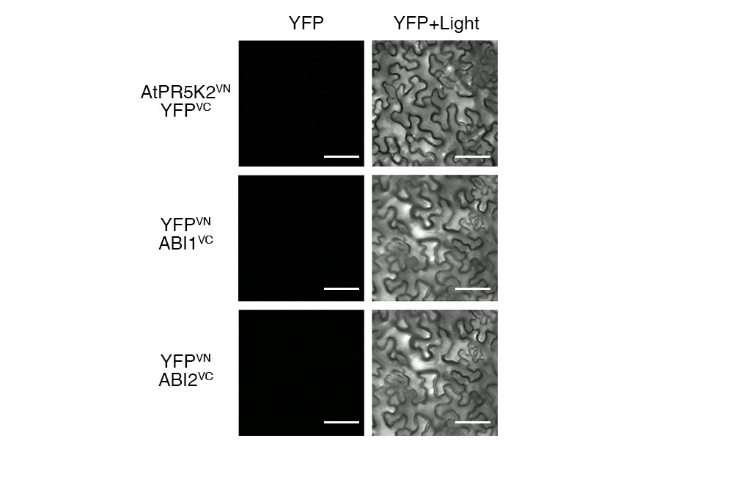


**Supplementary Figure 10.** Bimolecular fluorescence complementation (BiFC) assays between AtPR5K2, ABI1 or ABI2 and an empty vector.

BiFC analysis of empty vectors (YFP^VC^ and YFP^VN^) with AtPR5K2^VN^, ABI1^VC^ and ABI2^VC^ transiently co-expressed in tobacco (*Nicotiana Benthamiana*) leaves. VN and VC indicate the N- and C-terminal regions of Venus (eYFP), respectively. The epidermal cells were analyzed using confocal fluorescence microscopy and photographed after 48 hours of incubation at 25°C. Scale bars represent 100 μm.


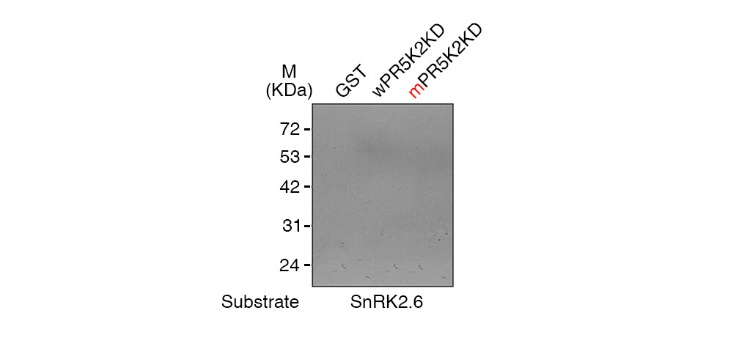


**Supplementary Figure 11.** In-gel kinase assay of AtPR5K2.

The recombinant fusion protein of the AtPR5K2 kinase domain (wPR5K2KD) or the mutagenized AtPR5K2 kinase domain (mPR5K2KD) was denatured and separated on an SDS gel. An in-gel kinase assay was performed using recombinant SnRK2.6 as a substrate for AtPR5K2 kinase domain. The GST tag served as a negative control.

**1. 2. Supplementary Table**

**Supplementary Table S1.** Primer lists for our study

| **Name** | **Sequences (5’→3’)** | **Purpose** |
| --- | --- | --- |
| *atpr5k1-1* LP | GTTCCAAATCCTCCTTTACCG | Mutants genotyping |
| *atpr5k1-1* RP | TCGTGGATGGTTACAACCTTC |  |
| *atpr5k2-1* LP | ATGACGAAGAGGTCGCCATTAATTC |  |
| *atpr5k2-1* RP | TTAAACCGCTTCCACAGTATATTGGT |  |
| *atpr5k3-1* LP | CACGTTTTGTCCGTCTAGCTC |  |
| *atpr5k3-1* RP | CCTTGCTCCAATCATCTCAAG |  |
| LBb1.3 | ATTTTGCCGATTTCGGAAC |  |
| GABI-Kat LB | CCCATTTGGACGTGAATGTAGACAC |  |
| attB1 adapter | GGGGACAAGTTTGTACAAAAAAGCAGGCT | Plasmid cloning |
| attB2 adapter | GGGGACCACTTTGTACAAGAAAGCTGGGT |  |
| AtPR5K2-attB1 | AAAAAGCAGGCTTAATGACGAAGAGGTCG |  |
| AtPR5K2-attB2-1 | AGAAAGCTGGGTCAACCGCTTCCACAGTA |  |
| AtPR5K2-attB2-2 | AGAAAGCTGGGTCTTAAACCGCTTCC |  |
| ABI1-attB1 | AAAAAGCAGGCTTAATGGAGGAAGTATCTCCG |  |
| ABI1-attB2 | AGAAAGCTGGGTCTCAGTTCAAGGGTTTGCTCT |  |
| ABI2-attB1 | AAAAAGCAGGCTTAATGGACGAAGTTTCTCCT |  |
| ABI2-attB2 | AGAAAGCTGGGTCTCAATTCAAGGATTTGCTCT |  |
| SnRK2.6-attB1 | AAAAAGCAGGCTTTATGGATCGACCAGTGAGT |  |
| SnRK2.6-attB2 | AGAAAGCTGGGTTTCACATTGCGTACACAATC |  |
| PYR1-attB1 | AAAAAGCAGGCTTTATGCCTTCGGAGTTAACA |  |
| PYR1-attB2 | AGAAAGCTGGGTTTCACGTCACCTGAGAACCA |  |
| AtPR5K2KD-F | GAATTCATGTTGAAACGTTATAGCTTTG | Site directed mutagenesis |
| AtPR5K2KD-R | GTCGACAACCGCTTCCACAGTATAT |  |
| mAtPR5K2KD-F | CGAGATATTGCTCTGAGAATCTTGAAA |  |
| mAtPR5K2KD-R | TGACTCTTTCAAGATTCTCAGAGCAAT |  |
| mABI1-F | GCCATGTCGAGAGCCATTGGCGATAGATAC TT |  |
| mABI1-R | AAGTATCTATCGCCAATGGCTCTCGACATGGC |  |
| mABI2-F | TCGCAATGTCAAGAGCCATTGGCGATAGATAC |  |
| mABI2-R | GTATCTATCGCCAATGGCTCTTGACATTGCGA |  |
| AtPR5K1-qRT-F | GACGCTTTACTCTACGAATTTCAAG | RT-PCR and qRT-PCR |
| AtPR5K1-qRT-R | CCTCTAGATTCCCCTCTAACATCTC |  |
| AtPR5K2-qRT-F | AGCCTCGAGTTCGGTTACAA |  |
| AtPR5K2-qRT-R | AAGCACGGTCGAAGTTCTGT |  |
| AtPR5K3-qRT-F | CACGTCCATCAATGTCAAGG |  |
| AtPR5K3-qRT-R | TGAGTTGGTGGAGCAGAGTG |  |
| TUBULIN2-F | AGCAAATGTGGGACTCCAAG |  |
| TUBULIN2-R | CACCTTCTTCATCCGCAGTT |  |
